# PTEN mediates Notch-dependent stalk cell arrest in angiogenesis

**DOI:** 10.1038/ncomms8935

**Published:** 2015-07-31

**Authors:** Helena Serra, Iñigo Chivite, Ana Angulo-Urarte, Adriana Soler, James D. Sutherland, Amaia Arruabarrena-Aristorena, Anan Ragab, Radiance Lim, Marcos Malumbres, Marcus Fruttiger, Michael Potente, Manuel Serrano, Àngels Fabra, Francesc Viñals, Oriol Casanovas, Pier Paolo Pandolfi, Anna Bigas, Arkaitz Carracedo, Holger Gerhardt, Mariona Graupera

**Affiliations:** 1 Vascular Signalling Laboratory, Institut d'Investigació Biomèdica de Bellvitge (IDIBELL), Gran Via de l'Hospitalet 199-203, 08908 L'Hospitalet de Llobregat, Barcelona, Spain; 2 CIC-bioGUNE, Technology Park of Bizkaia, 48160 Derio, Bizkaia, Spain; 3 Vascular Biology Laboratory, London Research Institute-Cancer Research UK, London WC2A 3LY, UK; 4 Max Planck Institute for Heart and Lung Research, D61231 Bad Nauheim, Germany; 5 Spanish National Cancer Research Center (CNIO), Madrid 28029, Spain; 6 UCL Institute of Ophthalmology, University Collage of London, London EC1V9EL, UK; 7 Centre d'Oncologia Molecular, IDIBELL, Universitat de Barcelona, 08907 L'Hospitalet de Llobregat, Barcelona,; 8 Translation Research Laboratory, Catalan Institute of Oncology, IDIBELL, Universitat de Barcelona, 08907 L'Hospitalet de Llobregat, Barcelona, Spain; 9 Departament de Ciències Fisiològiques II, Universitat de Barcelona, 08907 L'Hospitalet de Llobregat, Spain; 10 Cancer Research Institute, Beth Israel Deaconess Cancer Center, Department of Medicine and Pathology, Harvard Medical School, Boston, Massachusetts 02115, USA; 11 Program in Cancer Research, Hospital del Mar Medical Research Institute (IMIM), Barcelona Biomedical Research Park, 08003 Barcelona, Spain; 12 IKERBASQUE, Basque Foundation of Science, 48011 Bilbao, Bizkaia, Spain; 13 Department of Biochemistry and Molecular Biology, University of the Basque Country (UPV/EHU), Bilbao 48940, Spain; 14 Max-Delbrueck Center for Molecular Medicine (MDC), Robert-Rössle-Strasse 10, Berlin 13125, Germany

## Abstract

Coordinated activity of VEGF and Notch signals guides the endothelial cell (EC) specification into tip and stalk cells during angiogenesis. Notch activation in stalk cells leads to proliferation arrest via an unknown mechanism. By using gain- and loss-of-function gene-targeting approaches, here we show that PTEN is crucial for blocking stalk cell proliferation downstream of Notch, and this is critical for mouse vessel development. Endothelial deletion of PTEN results in vascular hyperplasia due to a failure to mediate Notch-induced proliferation arrest. Conversely, overexpression of PTEN reduces vascular density and abrogates the increase in EC proliferation induced by Notch blockade. PTEN is a lipid/protein phosphatase that also has nuclear phosphatase-independent functions. We show that both the catalytic and non-catalytic APC/C-Fzr1/Cdh1-mediated activities of PTEN are required for stalk cells' proliferative arrest. These findings define a Notch–PTEN signalling axis as an orchestrator of vessel density and implicate the PTEN-APC/C-Fzr1/Cdh1 hub in angiogenesis.

## Abstract (web summary)

During the formation of vascular sprouts, Notch activation inhibits proliferation of the stalk ECs via unknown mechanism. Here the authors show that PTEN represents a critical mediator of Notch anti-proliferative response in stalk cells via its phosphatase-dependent and -independent activity.

## Graphical abstract

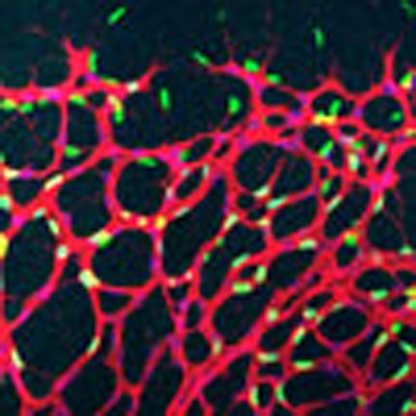

Vessel sprouting is a central mechanism of blood vessel growth^1^,^2^ and it relies on the induction of specialized endothelial cell (EC) populations, each accounting for distinct functions. At the very front of the sprouts, tip cells provide guidance and migrate towards gradients of vascular endothelial growth factor (VEGF)-A, but rarely proliferate^2^,^3^,^4^. Instead, trailing stalk cells located at the base of the sprout proliferate, establish adherent and tight junctions and form the vascular lumen^1^,^2^,^5^.

The tip cell phenotype is usually associated with high levels of Delta-like 4 (Dll4), which activate Notch in neighbouring stalk cells, preventing them from becoming a new tip cell. Notch signalling is initiated by receptor–ligand recognition between adjacent cells. This interaction results in two sequential proteolytic events that release the Notch intracellular domain (NICD). Subsequently, NICD translocates to the nucleus, where it forms a complex with the transcriptional factor Rbpj/Cbf1 and the Mastermind-like proteins to drive target gene expression^6^,^7^. Activation of Notch in ECs leads to cell cycle arrest both *in vitro*^8^ and *in vivo*^9^,^10^,^11^. However, it is still unclear how Notch exerts its negative effects on EC proliferation, and the transcriptional programme that triggers stalk cell function is not understood^2^,^5^,^12^. Furthermore, it is not clear how stalk cells are ultimately released from this arrest to provide sufficient cell numbers for the sprout to elongate and stabilize.

PTEN (phosphate and tensin homologue deleted on chromosome TEN) is a dual lipid/protein phosphatase, which is often underexpressed in cancer^13^,^14^,^15^. The main activity of PTEN is to dephosphorylate the lipid phosphatidylinositol 3,4,5-trisphosphate (PtdIns(3,4,5)P_3_) at the 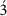-position, thereby counterbalancing class I phosphoinositide 3-kinase (PI3K) signalling that mediates growth, cell division, survival, migration and metabolism^13^,^16^,^17^,^18^. Genetic studies in mouse and zebrafish point to a restrictive role of PTEN in angiogenesis. Mice lacking PTEN specifically in ECs exhibit cardiac failure and severe haemorrhages due to defects of the myocardial wall and impaired mural cell coverage of blood vessels^19^. Mutant zebrafish embryos lacking functional PTEN show enhanced angiogenesis^20^; whether this is due to a cell-autonomous effect of PTEN in ECs or is simply a consequence of increased VEGF levels is unclear. Importantly, the specific functions of PTEN in endothelial behaviour and vascular patterning remain unknown.

In most cells and tissues, PTEN localizes to the cytoplasm and the nucleus^13^,^15^. There is evidence to suggest that PTEN has nuclear, non-lipid phosphatase-dependent functions^21^,^22^. Interestingly, PTEN localization is cell cycle-dependent, with higher levels of nuclear PTEN during the G0–G1 phase than during the S phase^23^,^24^. This is in line with the observation that nuclear PTEN negatively regulates cell cycle progression^13^,^22^. Indeed, in late mitosis and G1, nuclear PTEN enhances the E3 ligase activity of APC/C by facilitating the association of APC/C with its activator Fzr1/Cdh1 (encoded by the *Fzr1* gene), with no requirement of its phosphatase activity^22^. The APC/C-Fzr1/Cdh1 complex controls G1 progression by targeting several proteins for degradation, including mitotic cyclins (Cyclin-A), mitotic kinases (Aurora Kinase A (Aurora A) and Polo-like kinase 1 (Plk1)), proteins involved in chromosome segregation and DNA replication (Geminin; ref. 25). Despite the large body of molecular evidence, the role and relevance of nuclear PTEN in physiology is poorly understood.

Here we report that endothelial PTEN regulates stalk cell proliferation during vessel development. Our data further identify PTEN as a key mediator of the antiproliferative responses of Notch. We show that Dll4/Notch signalling arrests stalk cell proliferation by inducing expression of PTEN to balance stalk cell numbers and coordinate patterning. On PTEN deletion, Notch signalling fails to arrest early stalk cells and result in defective sprout length and patterning. Our results strongly indicate that both catalytic and non-catalytic activities of PTEN contribute to this function, providing evidence for an important *in vivo* physiological function for the PTEN-APC/C-Fzr1/Cdh1/axis.

## Results

### PTEN negatively regulates vascular density in angiogenesis

**Figure 1:**
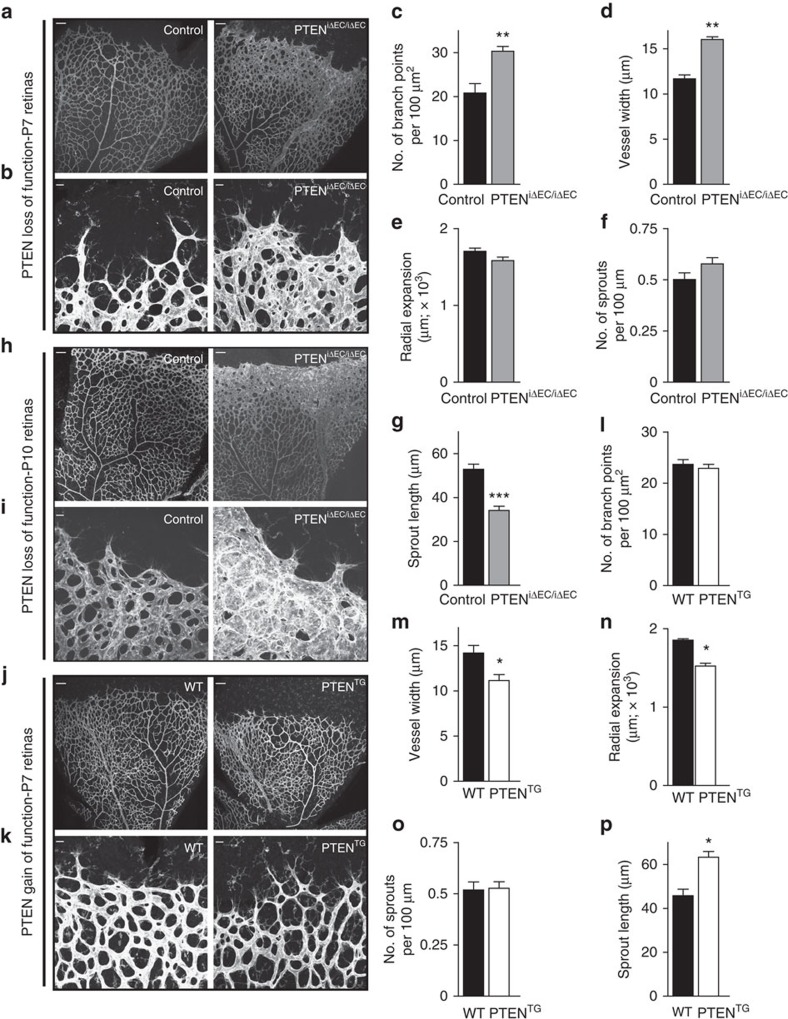
PTEN regulates vascular density. (**a**,**b**) Whole-mount visualization of blood vessels by isolectin B4 (IB4) staining of control and PTEN^iΔEC/iΔEC^ littermates at P7. (**c**–**g**) Quantitative analysis of the retinas shown in **a**,**b**. (**c**) Vascular branch points per unit area (*n*≥6). (**d**) Vessel width (*n*≥4). (**e**) Radial expansion of blood vessels (*n*≥7). (**f**) Number of sprouts per vascular front length (*n*≥7). (**g**) Sprout length from the tip to the base of the sprout (*n*≥7). (**h**,**i**) Whole-mount visualization of blood vessels by IB4 staining of control and PTEN^iΔEC/iΔEC^ littermates at P10. (**j**,**k**) Whole-mount visualization of blood vessels by IB4 staining of WT and PTEN^TG^ littermates at P7. (**l**–**p**) Quantitative analysis of the retinas shown in **j**,**k**. (**l**) Vascular branch points per unit area (*n*=8). (**m**) Vessel width (*n*=12). (**n**) Radial expansion of blood vessels (*n*=4). (**o**) Number of sprouts per vascular front length (*n*=6). (**p**) Sprout length from the tip to the base of the sprout (*n*=6). Scale bars, 100 μm (**a**,**h**,**j**) and 20 μm (**b**,**i**,**k**). Error bars are s.e.m. *P<0.05, **P<0.01 and ***P<0.001 were considered statistically significant. Statistical analysis was performed by nonparametric Mann–Whitney test.

To study the EC-autonomous role of PTEN in sprouting angiogenesis, we crossed *Pten*^*flox/flox*^ mice with *PdgfbiCreER*^*T2*^ transgenic mice that express a tamoxifen-activatable Cre recombinase in ECs^26^ (further referred to as PTEN^iΔEC/iΔEC^) and assessed postnatal retinal angiogenesis. 4-hydroxytamoxifen (4-OHT) was administrated *in vivo* at postnatal day 1 (P1) and P2, followed by analysis of the retinal vasculature at different time points. Comparing whole-mount-stained retinas of control (*Pten*^*flox/flox*^) to PTEN^iΔEC/iΔEC^ mice at P5 revealed a mild increase in vessel width (Supplementary Fig. 1a–g). By P7, loss of PTEN resulted in excessive branching and substantially increased vessel width (Fig. 1a–d), a phenotype that was further exacerbated at P10 (Fig. 1h,i). PTEN^iΔEC/iΔEC^ P7 retinas showed efficient recombination of the Cre-reporter R26-R and depletion of PTEN in the retinal endothelium (Supplementary Fig. 1h,i), with an increase in staining for phosphoS6 (pS6), a marker of PI3K pathway activation (Supplementary Fig. 1j). Isolated mouse lung ECs (mECs) from PTEN^iΔEC/iΔEC^ mice confirmed that effective depletion of PTEN protein in mECs was achieved 96 h following 4-OHT administration (Supplementary Fig. 1k,l). To further characterize the cell-autonomous role of PTEN in ECs, we therefore focused on the P7 time point. No differences in radial expansion (Fig. 1e) and in the number of sprouts per 100 μm of leading endothelial membrane were found in PTEN^iΔEC/iΔEC^ when compared with control retinas (Fig. 1f). Instead, the length of the sprouts was significantly reduced in the PTEN^iΔEC/iΔEC^ retinal vasculature compared with controls (Fig. 1g). The hyperplastic phenotype observed on PTEN loss was validated in an independent cellular system based on embryoid body (EB) formation, in which clusters of embryonic stem cells respond to VEGF by forming vascular tubes^27^. Compared with wild type (WT), PTEN null EBs showed increased sprout width and length (Supplementary Fig. 2a–d), with no differences in the number of sprouts (Supplementary Fig. 2e).

Next, we sought to address whether regulated elevation in PTEN expression *in vivo* would oppose the phenotype induced by loss of PTEN. To this end, we used super-PTEN transgenic mice (PTEN^TG^)^28^, a mouse model that allows moderate organismal elevation of PTEN levels (two-fold over WT littermates), including in the vasculature (Supplementary Fig. 1m). PTEN^TG^ retinas exhibited decreased vessel width and increased sprout length (Fig. 1j,k,m,p), with no changes in the number of branches (Fig. 1l) and sprouts (Fig. 1o). A slight reduction in radial expansion was also observed on moderate PTEN overexpression (Fig. 1n), similar to retinas from mice that are heterozygous for a kinase-dead p110α PI3K allele^29^. Neither loss nor gain of PTEN function resulted in changes in Dll4 or Notch target genes (Supplementary Fig. 3a–c), further supporting that PTEN is not required for tip/stalk selection. Analysis of *EphB4*, *EphrinB2* and *Nr2f2* gene expression, key genes involved in arteriovenous differentiation, did also not reveal any obvious difference between control and loss and gain of PTEN function in ECs, suggesting that PTEN signalling does not play a predominant role in this process (Supplementary Fig. 3d,e).

Taken together, these data uncover a selective role for PTEN in angiogenesis, regulating vascular density and consequently vessel growth *in vivo*.

### PTEN regulates endothelial stalk cell number

Previous data have shown that constitutive targeting of PTEN in ECs results in altered mural cell coverage^19^. Instead, immunostaining with desmin, a retinal pericyte marker^30^, did not reveal any obvious defect in mural cell coverage in PTEN^iΔEC/iΔEC^ retinas compared with control, consistent with the lack of sprouting defects on PTEN loss (Supplementary Fig. 3f).

**Figure 2:**
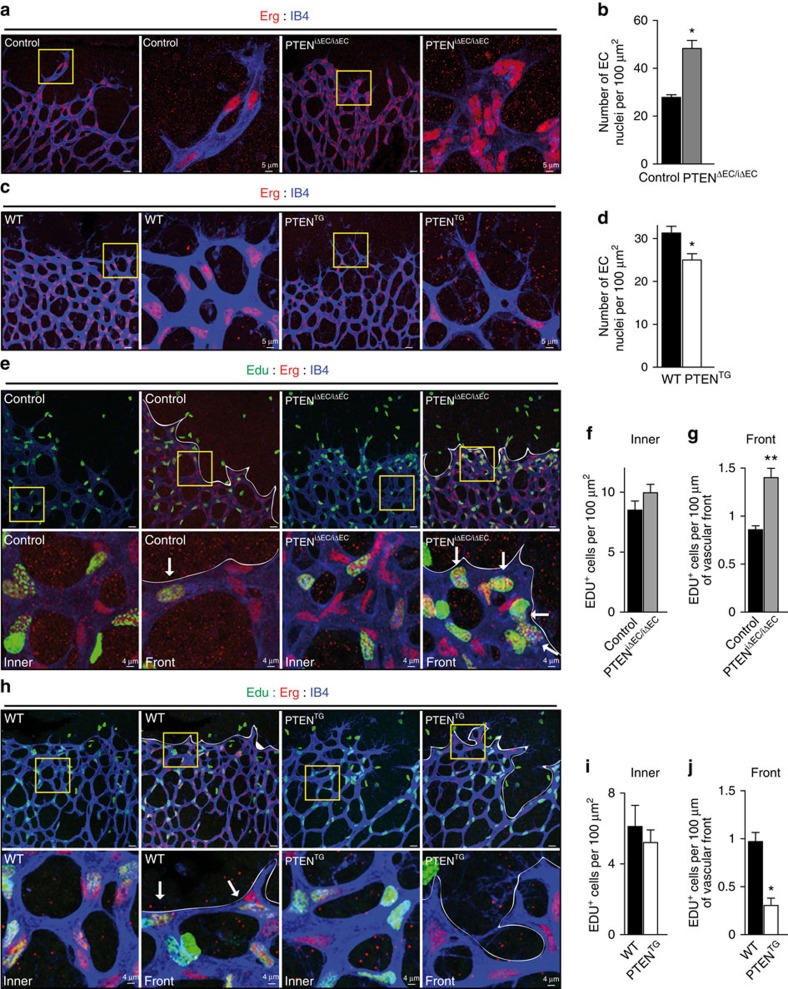
PTEN negatively regulates stalk cell proliferation. (**a**) IB4 (blue) and Erg (red) staining of control and PTEN^iΔEC/iΔEC^ littermate retinas at P7. Islets show higher magnification of selected regions shown to the right. (**b**) Quantification of EC nuclei per unit area assessed by Erg positivity in control and PTEN^iΔEC/iΔEC^ P7 retinas (*n*≥4). (**c**) IB4 (blue) and Erg (red) staining of WT and PTEN^TG^ littermate retinas at P7. Islets show higher magnification of selected regions shown to the right. (**d**) Quantification of EC nuclei per unit area assessed by Erg positivity in WT and PTEN^TG^ P7 retinas (*n*=8). (**e**) IB4 (blue), Erg (red) and Edu (green) staining of control and PTEN^iΔEC/iΔEC^ P7 retinas. Islets show higher magnification of selected regions shown below. Arrows indicate Edu-positive ECs in the sprouting front. (**f**) Quantification of Edu-positive cells per unit area assessed in control and PTEN^iΔEC/iΔEC^ P7 retinas (*n*≥7). (**g**) Quantification of number of Edu-positive cells located at the sprouting front expressed per vascular front length in control and PTEN^iΔEC/iΔEC^ P7 retinas (*n*=7). (**h**) IB4 (blue), Erg (red) and Edu (green) staining of WT and PTEN^TG^ littermate retinas at P7. Islets show higher magnification of selected regions shown below. Arrows indicate Edu-positive ECs in the sprouting front. (**i**) Quantification of Edu-positive cells per unit area assessed in WT and PTEN^TG^ P7 retinas (*n*=4). (**j**) Quantification of number of Edu-positive cells located at the sprouting front expressed per vascular front length in WT and PTEN^TG^ retinas (*n*=4). Scale bars, 20 μm (**a**,**c**,**e**,**h**). Error bars are s.e.m. *P<0.05 and **P<0.01 were considered statistically significant. Statistical analysis was performed by nonparametric Mann–Whitney test.

Analysis of PTEN^iΔEC/iΔEC^ retinas stained with a nuclear endothelial marker (Erg) revealed increased EC numbers in the angiogenic vasculature (Fig. 2a,b). Conversely, elevated PTEN expression resulted in reduced EC numbers at the sprouting front (Fig. 2c,d). We sought to validate whether these differences relate to changes in EC proliferation. Surprisingly, no difference in the number of proliferative ECs located in the subfront retinal area, behind the sprouting front, was found on either loss (Fig. 2e,f) or gain of PTEN function (Fig. 2h,i). This is unexpected, given that in the growing vasculature ECs with high turnover are located in this subfront area (Supplementary Fig. 4a,b). To test whether PTEN regulated proliferation of ECs in other retinal locations, we focused on the first line of cells located at the sprouting front where proliferating cells are rarely observed (Supplementary Fig. 4a,b). Interestingly, a 40% increase in proliferation in PTEN^iΔEC/iΔEC^ retinas (Fig. 2g) or 60% reduction in PTEN^TG^ retinas (Fig. 2j) compared with control retinas was observed in ECs at the front. These data point towards a selective role of PTEN in restricting EC proliferation in cells located at the sprouting front.

### PTEN executes Notch-dependent cell cycle arrest

**Figure 3:**
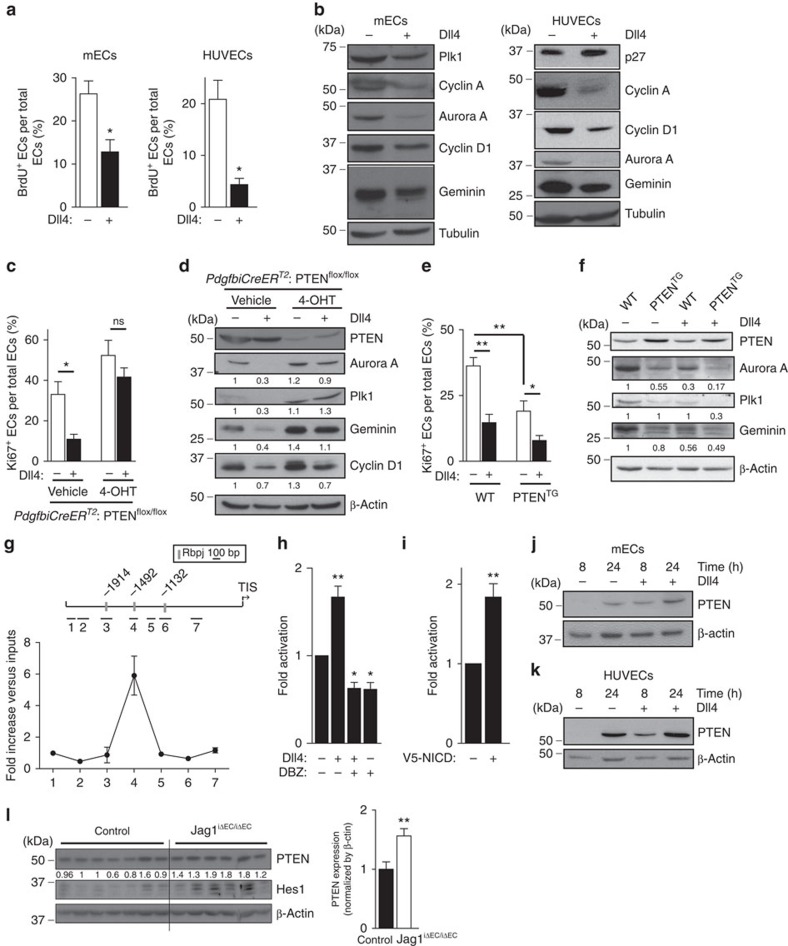
Notch limits EC proliferation by upregulating PTEN levels. (**a**) Quantification of *in vitro* proliferation of mECs and HUVECs plated for 24 h in vehicle or Dll4-coated dishes, pulsed with BrdU for 2 h and subjected to immunostaining analysis. At least 100 cells per condition were counted (*n*=4). (**b**) Immunoblot analysis of mECs and HUVECs plated for 24 h in vehicle or Dll4-coated plates using the indicated antibodies (*n*=3). Molecular weight marker (kDa) is indicated. (**c,e**) Quantification of *in vitro* proliferation by Ki67 immunofluorescence of control and PTEN^iΔEC/iΔEC^ (**c**) and WT and PTEN^TG^ mEC (**e**) plated for 24 h in vehicle or Dll4-coated plates. Overall 100 cells per condition were counted (*n*=4). (**d**) Control and PTEN^iΔEC/iΔEC^ or (**f**) WT and PTEN^TG^ mEC were plated for 24 h in vehicle or Dll4-coated dishes, followed by immunoblot analysis using the indicated antibodies. The quantification of the relative immunoreactivity of each protein normalized to β-actin is represented as the mean of four different experiments in **d**,**f**. Molecular weight marker (kDa) is indicated. (**g**) ChIP with the anti-NICD antibody from HUVECs and the analysis of the PTEN locus by qPCR. A pool of two independent experiments is shown. (**h**,**i**) PTEN-luciferase reporter assays were performed in HUVECs with a 2,666-bp *PTEN* promoter construct (pGL3 PTEN *Hind III-NotI*). (**h**) Cells were plated for 6 h in vehicle or Dll4-coated dishes and (**i**) HUVECs were transfected with V5-NICD (*n*=3). (**j**,**k**) Immunoblot analysis of PTEN in lung mECs (**j**) and in HUVECs (**k**) plated for 8 or 24 h in vehicle or Dll4-coated plates (*n*=4). Molecular weight marker (kDa) is indicated. (**l**) Immunoblot analysis of PTEN and Hes in lung lysates from control (*n*=7) and Jag1^iΔEC/iΔEC^ (*n*=6) pups. Molecular weight marker (kDa) is indicated. Error bars are s.e.m. *P<0.05 and **P<0.01 were considered statistically significant. ns, not statistically significant. Statistical analysis was performed by nonparametric Mann–Whitney test.

Given that the impact of PTEN loss or overexpression *in vivo* on proliferation are restricted to the sprouting front that is highly Notch-dependent^9^, we hypothesized that a functional connection exists between these two signalling pathways. Activation of Notch in mECs, both *in vivo* and *in vitro*, resulted in cell cycle arrest, shown by reduced 5-bromo-2'-deoxyuridine (BrdU) incorporation (Supplementary Fig. 4c,d and Fig. 3a) and downregulation of cell cycle regulators including Cyclin D and A, Plk1, Aurora A and Geminin (Fig. 3b). Interestingly, genetic manipulation of PTEN levels in ECs altered the antiproliferative response to Notch activation (Fig. 3c,e). PTEN^iΔEC/iΔEC^ mECs failed to stop proliferation on Notch activation (Fig. 3c,d), whereas PTEN^TG^ mECs showed a 50% increase in the cell cycle arrest response (Fig. 3e,f). Consistent with a role of PTEN in regulating the cell cycle arrest, higher PTEN levels in ECs *in vivo* corresponded to nonproliferative cells (Supplementary Fig. 4e,f). Our results suggest that PTEN is necessary for the growth-suppressive activity of Notch signalling in ECs and imply that the PTEN loss-of-function phenotype is the result of an impaired response to Dll4 stimulation.

Because PTEN is required for the Notch-dependent regulation of endothelial proliferation, we tested whether PTEN expression is regulated by Notch. Bioinformatic analysis of the *PTEN* locus identified the presence of three Rbpj motifs that are conserved in both the human and mouse *PTEN* gene (Fig. 3g). We used chromatin immunoprecipitation (ChiP) analysis on human ECs to determine the recruitment of NICD protein to the *PTEN* promoter after 2 h of incubation with Dll4. PTEN promoter occupancy was determined using real-time quantitative PCR (qPCR) probes that amplify seven regions spanning from −2,380 to −590 relative to the transcription initiation site. Our analysis revealed NICD occupancy in the −1,492 region, which contains one of the three predicted Rbpj-binding sites (Fig. 3g). We next validated the functional significance of Rbpj binding to the promoter in luciferase reporter assays. Indeed, analysis of PTEN promoter activity showed activation in response to Dll4 (Fig. 3h) and on overexpression of the intracellular domain of the Notch receptor (NICD; Fig. 3i). Importantly, enhanced PTEN promoter responsiveness to Dll4 was abrogated by the γ-secretase inhibitor dibenzazepine (DBZ; Fig. 3i). Western blot experiments confirmed that Dll4 stimulation results in elevated protein levels of PTEN in human ECs and mECs (Fig. 3j,k). Using lungs as highly vascularized tissue, we validated that overactivation of Notch signalling by inhibiting the Notch ligand, Jagged 1 (Jag1)^11^, resulted in higher PTEN expression levels (Fig. 3l). Taken together, these results demonstrate that PTEN is a target gene of Notch signalling in ECs, which becomes induced by Dll4 stimulation.

### PTEN is required for Notch function *in vivo*

**Figure 4:**
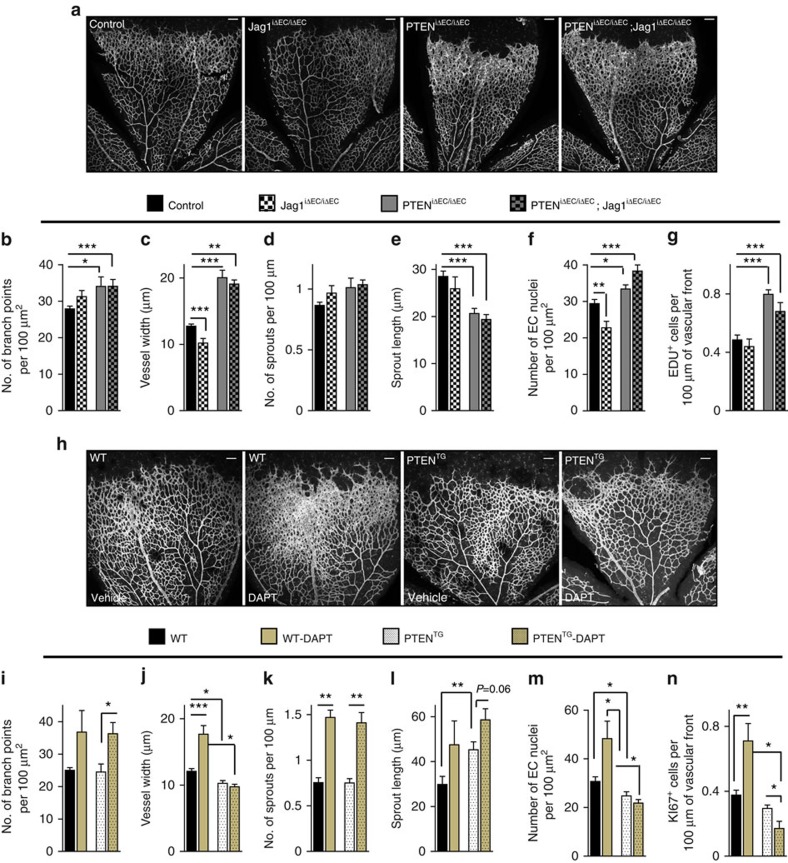
PTEN interacts with Notch *in vivo* to negatively control stalk cell proliferation. (**a**) Whole-mount visualization of blood vessels by IB4 staining of control, Jag1^iΔEC/iΔEC^, PTEN^iΔEC/iΔEC^ and PTEN^iΔEC/iΔEC^; Jag1^iΔEC/iΔEC^ littermates at P7. (**b**–**g**) Quantitative analysis of the retinas shown in **a**. Retinas from five independent litters were pooled for quantification. (**b**) Vascular branch points (*n*≥4). (**c**) Vessel width (*n*≥4). (**d**) Number of sprouts per vascular front length (*n*≥4). (**e**) Sprout length from the tip to the base of the sprout (*n*≥4). (**f**) Quantification of EC nuclei per unit area assessed by Erg positivity (*n*≥4). (**g**) Quantification of number of Edu-positive cells located at the vascular front expressed per sprouting front length (*n*≥3). (**h**) Whole-mount visualization of blood vessels by IB4 staining of WT and PTEN^TG^ P7 retinas treated with vehicle or DAPT (100 mg kg^−1^). (**i**–**n**) Quantitative analysis of the retinas shown in **h**. Retinas from three independent litters were pooled for quantification. (**i**) Vascular branch points per unit area (*n*≥4). (**j**) Vessel width (*n*≥4). (**k**) Number of sprouts per vascular front length (*n*≥4). (**l**) Sprout length from the tip to the base of the sprout (*n*≥4). (**m**) Quantification of endothelial nuclei per unit area assessed by Erg positivity (*n*≥4). (**n**) Quantification of number of Ki67-positive cells located at the vascular front expressed per sprouting front length (*n*=6). Scale bars, 100 μm (**a**,**h**). Error bars are s.e.m. *P<0.05, **P<0.01 and ***P<0.001 were considered statistically significant. Statistical analysis was performed by nonparametric Mann–Whitney test.

We sought to confirm the Notch/PTEN functional interaction *in vivo*. We took advantage of endothelial Jag1 inactivation, which results in reduced EC proliferation and decreased vascular branching due to overactivation of Notch signalling^11^. We hypothesized that, if the regulation of the angiogenic process by Notch requires the increase in PTEN function, loss of PTEN would prevent the phenotype of Jag1 deletion. We tested this hypothesis in inducible endothelial-cell-specific PTEN^iΔEC/iΔEC^;Jag1^iΔEC/iΔEC^ double mutants. The vasculature of Jag1^iΔEC/iΔEC^ retinas showed reduced endothelial proliferation and reduced vessel width, confirming previous reports (Fig. 4a–g and Supplementary Fig. 5a)^11^. However, concomitant PTEN deletion abrogated the phenotype observed in Jag1^iΔEC/iΔEC^ retinas (Fig. 4a–g), while the phenotype of PTEN loss remained unaffected by Jag1 deletion (the increase in endothelial proliferation at the spouting front). Next, we validated our hypothesis in the PTEN^TG^ mice by blocking Notch activation with the γ-secretase inhibitor DAPT (N-[N-(3,5-difluorophenacetly)-L-alanyl]-S-phenylglycine t-butyl ester) that strongly enhances angiogenesis partially due to increased EC proliferation^9^. Remarkably, DAPT-induced increase in vascular density at the angiogenic front was abolished by increased levels of PTEN (Fig. 4h,j,m,n and Supplementary Fig. 5b). However, elevated levels of PTEN did not prevent the enhanced sprouting caused by DAPT (Fig. 4i,k,l), further indicating that PTEN is not required for Notch-dependent tip/stalk selection.

### Dual function of PTEN in angiogenesis

**Figure 5:**
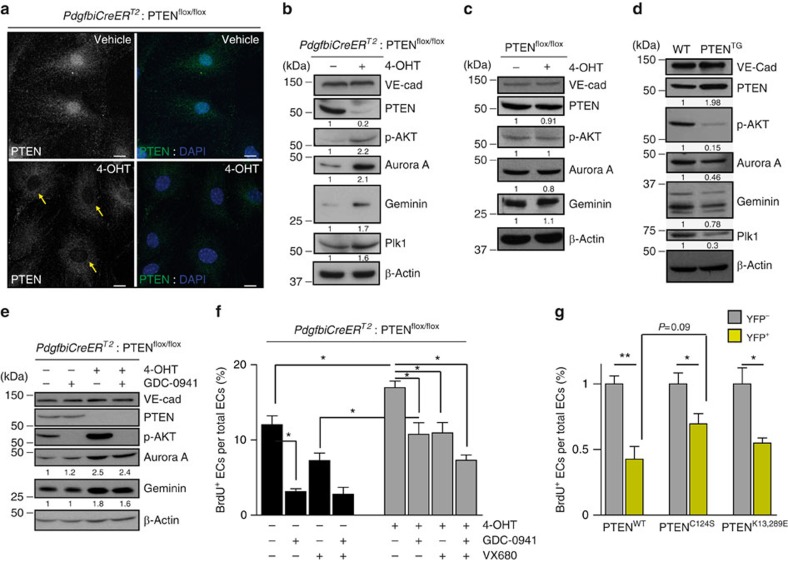
Catalytic and non-catalytic roles of PTEN regulate EC proliferation. (**a**) Confocal images of PTEN (green) and DAPI immunofluorescence in *PdgfbiCreER*^*T2*^; PTEN^flox/flox^ mECs treated with vehicle or with 4-OHT for 96 h. Yellow arrows indicate the lack of PTEN staining in the nucleus of PTEN null cells. Scale bars, 10 μm (*n*=3). (**b**–**d**) Exponentially growing mECs were lysated, followed by immunoblotting using the indicated antibodies. *PdgfbiCreER*^*T2*^; PTEN^flox/flox^ (**b**) and PTEN^flox/flox^ (**c**) mECs were treated for 96 h with vehicle or 4-OHT. (**d**) WT and PTEN^TG^ mECs were cultured for 48 h before cell lysis and immunoblotting. (**e**) *PdgfbiCreER*^*T2*^; PTEN^flox/flox^ mECs were treated for 96 h with vehicle or 4-OHT. Before cell lysis, cells were pretreated for 2 h with GDC-0941 (1 μM). The quantification of the relative immunoreactivity of each protein normalized to β-actin is represented as the mean from at least three different experiments in **b**–**e**. Molecular weight marker (kDa) is indicated. (**f**) Exponentially growing control and PTEN^iΔEC/iΔEC^ mECs were treated for 48 h with test compounds or vehicle, and then were pulsed with BrdU for 2 h and subjected to immunostaining analysis. Inhibitors and doses used were as follows: GDC-0941 (pan-class I PI3K inhibitor; 1 μM) and VX680 (Aurora Kinase inhibitor; 0.5 μM). Data shown are means of four independent experiments. (**g**) *PdgfbiCreER*^*T2*^; PTEN^flox/flox^ mECs were infected with PTEN^WT^, PTEN^C124S^ or PTEN^K13,289E^, treated with 4-OHT for 72 h, plated for 48 h in the presence of doxycycline, pulsed with BrdU for 2 h and subjected to immunostaining analysis. Data shown are the means of six independent experiments. Error bars are s.e.m. *P<0.05 and **P<0.01 were considered statistically significant. Statistical analysis was performed by nonparametric Mann–Whitney test.

To gain insight into the biological mechanism underlying the role of PTEN in sprouting angiogenesis, we investigated the contribution of phosphatase-dependent and -independent activities of PTEN at the organismal and cellular levels. We observed a compartmentalization of PTEN in the nucleus and cytoplasm in cultured control cells (Fig. 5a), whereas PTEN^iΔEC/iΔEC^ mECs showed no nuclear staining with some residual positivity in the cytoplasm. PTEN depletion in mECs resulted in increased Akt phosphorylation and in accumulation of the E3 ubiquitin ligase APC/C-Fzr1/Cdh1complex substrates Aurora A, Plk1 and Geminin (Fig. 5b). Control mECs treated with 4-OHT did not show any of the aforementioned changes (Fig. 5c). In contrast, mECs isolated from PTEN^TG^ lungs showed reduced Akt phosphorylation and reduced accumulation of E3 ubiquitin ligase APC/C-Fzr1/Cdh1 complex substrates compared with WT cells (Fig. 5d).

To investigate whether increased levels of the APC/C-Fzr1/Cdh1 targets were only a consequence of increased PI3K activity, we tested the impact of GDC-0941 (a pan-class I PI3K inhibitor that blocks p110α, p110β, p110δ and p110γ; ref. 31). While pretreatment with GDC-0941 abrogated Akt activation in PTEN^iΔEC/iΔEC^ mECs (Fig. 5e), it did not modify the levels of Aurora A and Geminin (Fig. 5e), further corroborating that the function of PTEN promoting the APC/C-Fzr1/Cdh1 activity is independent of its ability to inhibit PI3K signalling through its lipid phosphatase activity^22^.

As our data indicate that the principal function of PTEN in sprouting angiogenesis is to regulate EC proliferation, we tested to what extent phosphatase-dependent and -independent activities of PTEN participate in this regulation. *In vitro* isolated PTEN^iΔEC/iΔEC^ and PTEN^TG^ mECs showed increased and decreased BrdU incorporation, respectively (Fig. 5f and Supplementary Fig. 6a,b). Inhibition of PI3K activity and Aurora kinase, one of the main targets of APC/C-Fzr1/Cdh1 (ref. 22), partially rescued normal proliferation rate in PTEN^iΔEC/iΔEC^ mECs (Fig. 5f and Supplementary Fig. 6a). A synergistic effect was observed on pretreatment with both GDC-0941 and VX680, further implying a dual function of PTEN in this process. Next, we complemented PTEN-depleted ECs with either WT (PTEN^WT^), phosphatase-inactive (C124S; PTEN^C124S^; refs 32, 33) or nuclear-excluded (K13,289E; PTEN^K13,289E^) PTEN (ref. 22). Expression of PTEN^WT^, PTEN^C124S^ and PTEN^K13,289E^ abrogated the increase in EC proliferation (Fig. 5g and Supplementary Fig. 6c) of PTEN null ECs, albeit most prominently seen with PTEN^WT^. All together, these data indicate that phosphatase-dependent and -independent activities of PTEN are important to regulate EC proliferation.

**Figure 6:**
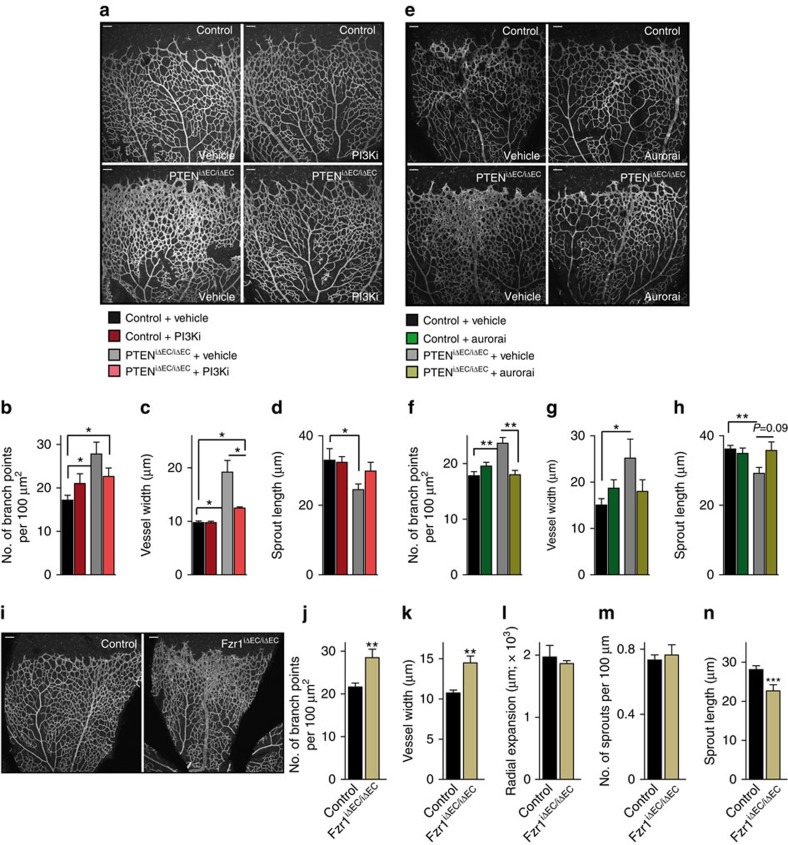
Dual function of PTEN in sprouting angiogenesis. (**a**) IB4-stained control and PTEN^iΔEC/iΔEC^ P7 retinas (4-OHT administration from P1 to P2) treated with vehicle or GDC-0941 at P6 and P7. (**b**–**d**) Quantitative analysis of the retinas shown in **a**. (**b**) Vascular branch points per unit area (*n*≥4). (**c**) Vessel width (*n*≥4). (**d**) Sprout length from the tip to the base of the sprout (*n*≥4). (**e**) IB4-stained control and PTEN^iΔEC/iΔEC^ P7 retinas (4-OHT administration from P1 to P2) treated with vehicle or VX680 at P6 and P7. (**f**–**h**) Quantitative analysis of the retinas shown in **e**. (**f**) Vascular branch points per unit area (*n*≥6). (**g**) Vessel width (*n*≥6). (**h**) Sprout length from the tip to the base of the sprout (*n*≥6). (**i**) Overview of P7 control and Fzr1^iΔEC/iΔEC^ iB4-stained. (**j**–**n**) Quantitative analysis of the retinas shown in **i**. (**j**) Vascular branch points per unit area (*n*=11). (**k**) Vessel width (*n*=11). (**l**) Radial expansion of blood vessels (*n*≥5). (**m**) Number of sprouts per vascular front length (*n*=11). (**n**) Sprout length from the tip to the base of the sprout (*n*=6). Scale bars, 100 μm (**a**,**e**,**i**). Error bars are s.e.m. *P<0.05, **P<0.01 and ***P<0.001 were considered statistically significant. Statistical analysis was performed by nonparametric Mann–Whitney test.

Next, we tested the differential contribution of each of these functions *in vivo*, by first analysing the retinas of PTEN^iΔEC/iΔEC^ on inhibition of class I PI3K activity with GDC-0941. The hyperplasia induced by PTEN loss was rescued by inhibiting PtdIns(3,4,5)P_3_ production (Fig. 6a–d and Supplementary Fig. 7a), indicating, as expected, an important role of PTEN lipid phosphatase activity in vascular growth. Next, we assessed the contribution of phosphatase-independent activity of PTEN to sprouting angiogenesis by inhibiting Aurora kinase. Strikingly, the phenotype of PTEN^iΔEC/iΔEC^ was abrogated by Aurora kinase inhibition (Fig. 6e–h), which is consistent with the contribution of the phosphatase-independent activity of PTEN to sprouting angiogenesis *in vivo*. This was further corroborated by genetic conditional and inducible deletion of *Fzr1* in ECs. Complete depletion of Fzr1/Cdh1 protein was achieved 48 h post incubation with 4-OHT (Supplementary Fig. 7b,c). Therefore, pups were treated with 4-OHT at P5 and P6, followed by analysis of the retinal vasculature at P7. The retinas of Fzr1^iΔEC/iΔEC^ showed increased vascular density and reduced length of sprouts (Fig. 6i–n) comparable to PTEN^iΔEC/iΔEC^. All together, these data suggest that, in ECs, there is a dual contribution of PTEN, counterbalancing the PI3K signalling pathway through its lipid phosphatase activity and facilitating the APC/C-Fzr1/Cdh1 complex activity, which has been reported to be independent of its catalytic function^22^.

## Discussion

Here we report that PTEN in ECs is required in a cell-autonomous and dose-dependent manner for the control of vascular density and vessel growth, but is dispensable for the regulation of the sprouting activity of tip cells. We show that endothelial PTEN restricts vascular growth by limiting stalk cell proliferation during sprouting angiogenesis. An important conclusion from this study is that PTEN is not required in all ECs to regulate proliferation *in vivo*, as shown in cultured ECs (our data and ref. 19). Instead, our results indicate that the consequence of PTEN loss or overexpression *in vivo* is restricted specifically to the zone of the vasculature that is highly Notch-dependent.

Constitutive targeting of PTEN in ECs leads to embryonic lethality due to aberrant angiogenesis^19^. Although these studies have established that PTEN regulates EC proliferation, the analysis of vasculature in Tie2Cre-PTEN^flox/flox^ embryos did not allow unravelling precisely how PTEN regulates sprouting angiogenesis. Furthermore, Hamada *et al*. were unclear of whether the aberrant vasculature, which results on PTEN loss, was a consequence of aberrant PTEN signalling in ECs or simply a consequence of an altered pro-angiogenic cytokine profile^19^. Similarly, zebrafish studies have shown that loss of PTEN leads to increased VEGF levels and in turn vessel hyperplasia, highlighting indeed that PTEN also regulates angiogenesis in a paracrine manner^20^. Another difference between Hamada *et al*. and our study is that constitutive loss of PTEN in ECs also results in altered mural cell coverage, while induced loss of postnatal endothelial PTEN does not. These discrepancies suggest that PTEN may differently regulate angiogenesis in different vascular beds.

Activation of Notch leads to cell cycle arrest^8^,^9^,^10^,^11^,^34^. In this study, we identify PTEN as a critical mediator of Notch antiproliferative response in stalk cells. If PTEN is not expressed in ECs, stalk cells become insensitive to the antiproliferative signals of Notch and exhibit unrestricted expansion, hence perturbing sprout length and pattern and eventually resulting in profound hyperplasia. Interestingly, our results also reveal that stalk cells located further away from the front are insensitive to changes in PTEN expression. Given that these stalk cells at the subfront area are highly proliferative, our data support the existence of two biological states for stalk cells. A first state in which stalk cells must remain arrested to ensure the correct patterning of the sprout, and a second state in which cells enter the cell cycle to expand the plexus. These two states are likely the consequence of dynamic changes in Notch signalling, with high Notch activity in early nonproliferative stalk cells and low Notch activity in the late proliferative stalk cells. Our data predict a rise and fall in PTEN levels that will accompany the early quiescent and late proliferative phases, respectively. This is supported by the observation that, in WT cultured ECs, higher PTEN levels are seen 8 h post stimulation with Dll4 compared with 24 h post stimulation. Furthermore, co-staining of PTEN and 5-ethynyl-2′-deoxyuridine (Edu) in the growing vasculature showed that Edu-negative cells express higher levels of PTEN than Edu-positive cells, supporting the notion that PTEN protein levels rise to guarantee cell cycle arrest. Whether high PTEN cells correspond to high Notch signalling still needs to be determined. Moya *et al*. speculated that early and late stalk cell behaviours might be orchestrated by oscillation in Notch activity. The authors proposed that Id proteins, members of HLH proteins, govern these two states by releasing the negative autoregulatory loop of Hes1 (ref. 35). While our results are consistent with the idea of two states, they identify PTEN as the key mediator of early stalk cell function in response to Notch.

Why and how Notch exerts a unique negative regulation in the endothelium while driving proliferation in virtually every other cell type and in cancer has been a mystery^5^,^36^. Our data show that PTEN negatively regulates cell cycle progression in ECs through conserved pathways. Critically, what our results illustrate is a novel interaction between Notch and PTEN in ECs. We find that Notch stimulates PTEN transcription in the endothelium, an effect that is required for Notch-mediated cell cycle arrest. Interestingly, in cell types where Notch stimulates cell cycle progression, PTEN is transcriptionally repressed by Notch/Hes^36^,^37^,^38^. The PTEN gene locus contains both Rbpj- and Hes-binding sites, suggesting that binding to one or another is what determines the final biological output.

In line with the observation that PTEN restricts stalk cell proliferation, endothelial gain and loss of PTEN proliferation phenotypes are reminiscent of gain and loss of Notch function in stalk cells^9^,^10^,^11^. However, in response to Notch signalling PTEN appears to only regulate EC proliferation while it is not required for tip and stalk specification. This is shown by the observation that Notch mutants not only show aberrant proliferation phenotypes in the nascent plexus but also sprouting defects^9^,^11^, while PTEN mutants only show vascular density defects. In the same line, increased levels of PTEN protect angiogenic ECs treated with DAPT from uncontrolled proliferation but fail to prevent excessive tip cell numbers. Conversely, a recent study has shown that inhibitors of the VEGFR3 kinase activity rescue the hypersprouting phenotype of Notch loss-of-function mutants, without reducing EC proliferation^39^. Taken together, these data suggest that Notch regulates tip cell numbers and stalk cell proliferation independently through VEGFR3 and PTEN pathways, respectively.

The predominant activity of PTEN is the dephosphorylation of PtdIns(3,4,5)P_3_ and thus the counteraction of class I PI3K-mediated functions^13^,^15^. However, PTEN also exhibits PtdIns(3,4,5)P_3_-independent functions, including protein phosphatase^14^ and non-catalytic activities^13^,^15^. In this context, PTEN can be found in the nucleus where it regulates DNA stability and cell cycle progression^22^,^40^. Several reports have highlighted the relevance of nuclear PTEN in disease^22^,^41^,^42^,^43^. To date, the physiological relevance of nuclear PTEN *in vivo* remains elusive. Our results reveal that both lipid phosphatase-dependent and non-catalytic activities of PTEN regulate stalk cell proliferation during sprouting angiogenesis. Inhibition of class I PI3K activity with GDC-0941 or Aurora kinase with VX680 significantly abrogates the phenotype observed on PTEN loss. However, the observation that pretreatment with either GDC-0941 or VX680 is not able to completely rescue the hyperplasia phenotype of PTEN^iΔEC/iΔEC^ retinas and cultured ECs indicates that both types of activities of PTEN are required to drive the PTEN response in angiogenesis. Furthermore, genetic deletion of *Fzr1* in ECs recapitulates the phenotype observed on endothelial loss of PTEN, reinforcing the relevance of nuclear PTEN facilitating the APC/C-Fzr1/Cdh1 function. Taken together, our study provides *in vivo* evidence that nuclear PTEN is not only involved in disease such as cancer or cerebral ischaemia^22^,^41^,^42^,^43^ but is also critical to regulate a fundamental physiological process such as angiogenesis.

We and others have previously shown that inhibition of class I PI3K isoform *in vivo* does not lead to blockade of EC proliferation^29^,^44^,^45^,^46^. Although contradictory, these observations may reflect that PTEN principally regulates EC proliferation independently of its lipid phosphatase activity^22^. In line with this, our data also reveal that the regulation of APC/C-Fzr1/Cdh1 by PTEN seems to play a major role in response to Notch signalling in angiogenesis. This is shown by the altered APC/C-Fzr1/Cdh1 target expression under conditions of PTEN loss and Notch activation. This observation, together with the fact that Notch stimulation in ECs results in phosphorylation of Akt^47^,^48^, suggest that Notch stimulates PTEN nuclear translocation. These findings would be in agreement with the notion that higher nuclear PTEN levels are found during G0–G1 phase than during the S phase^23^,^24^. Further experiments are needed to elucidate how PTEN accumulates in the nucleus on Notch activation.

**Figure 7:**
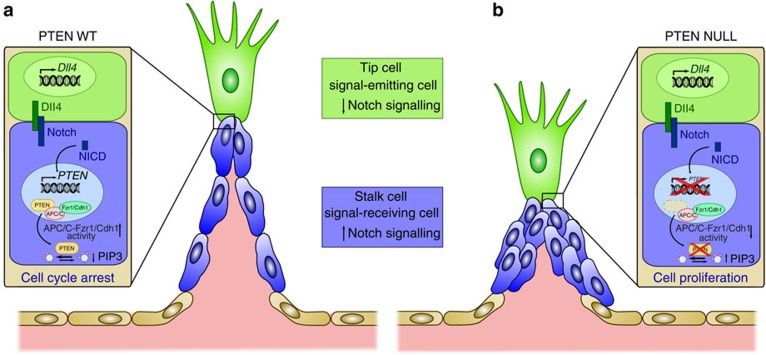
Schematic model of the role of PTEN in Dll4/Notch-mediated stalk cell cycle arrest. (**a**) Activation of Notch by Dll4 induces expression of PTEN, which through its lipid phosphates activity and its nuclear function as a scaffold of the APC/C-Fzr1/Cdh1 blocks stalk cell proliferation. (**b**) On PTEN loss, Notch signalling fails to arrest stalk cells and result in defective sprout length and patterning.

The unique direction of the coupling of Notch and PTEN in the endothelium (Fig. 7), and the highly selective effects on the active vascular front raise the prospect that targeting this interaction and stimulation of PTEN signalling may be used therapeutically to render EC quiescence in aberrant tumour angiogenesis and in turn promote a normalization effect. Clinically, our results imply that stimulating both arms of PTEN function in ECs could render a more quiescence phenotype of highly proliferative tumour ECs^2^,^49^. However, inhibition of PI3K in the tumour stroma not only results in reduced EC proliferation but also in reduced vascular function^47^. It is thus tempting to speculate that promoting nuclear PTEN may offer more selectivity towards a tight control of EC proliferation.

## Methods

### Reagents

Sources and catalogue numbers of antibodies were as follows: Cell Signaling Technology: PTEN (#9559), pS473-Akt (#4060) and pSer240/244-S6 (#2215); BD Pharmingen: p27 (#610242), cyclin-D1 (#556470), BrdU (#347580) and Aurora A Kinase (#610939); NeoMarkers: Ki67 (#RM-9106-S); Abcam: NICD (#ab27526), desmin (ab15200) and PTEN (ab32199); Santa Cruz Biotechnology: VE-cadherin (sc-6458), Geminin (#sc-13015), cyclin-A (#sc-53230), Hes1 (#sc-25392) and Erg (sc-353); Millipore: Plk1 (#06-813) and Fzr1/Cdh1 (#CC43); Sigma-Aldrich: β-actin (A5441) and α-tubulin (T6074). Isolectin GS-IB_4_ and secondary antibodies conjugated to Alexa 488, Alexa 568 and Alexa 633, and Click-iT EdU Alexa 488 and 647 Imaging Kit were from Molecular Probes. Human (#1506-D4) and mouse Dll4 (#1389-D4) were from R&D Systems. The GDC-0941 compound was from Chem Express Haoyuan (China). The VX680 (MK-0457) compound was from Selleckchem (USA). All chemicals, unless otherwise stated, were from Sigma-Aldrich.

### Inducible genetic protocols and pharmacological inhibition in mice

Mice were kept in individually ventilated cages and cared for according to the guidelines and legislation of the UK Home Office and Catalan Departament d' Agricultura, Ramaderia i Pesca, with procedures accepted by the Ethics Committees of CRUK-London Research Institute and IDIBELL-CEEA.

To delete PTEN in postnatal vessels, we crossed the PTEN^flox^ mice^50^ into the transgenic mice expressing the tamoxifen-inducible recombinase CreER^T2^ under the control of the endothelial *Pdgfb* promoter^26^. To generate *PdgfbiCreER*^*T2*^; PTEN^flox/flox^ and PTEN^flox/flox^ littermates, *PdgfbiCreER*^*T2*^; PTEN^flox/flox^ were interbred with PTEN^flox/flox^. Cre activity and gene deletion were induced by intraperitoneal injection of 25 μg 4-OHT (Sigma, H7904 10 mg ml^−1^) in all pups of the litter at P1 and P2, and retinas were collected at different time points (P5, P7 and P10). Class I PI3K signalling or Aurora kinase was inhibited in half of the pups by subcutaneous injection at 18:00 pm of P6 and 10:00 am of P7 with 37.5 μg g^−1^ GDC-0941 (ref. 31) or with 50 μg g^−1^ VX680, respectively, dissolved in dimethylsulphoxide (DMSO). Retinas were harvested at 18:00 pm of P7. Control mice were injected with DMSO only.

PTEN^TG^ (ref. 28) was maintained in C57/BL6 background and were fed with a 19% protein-extruded rodent diet (Harlan, 2019) in a 1:1 proportion with normal diet. Notch signalling was inhibited in half of the pups by subcutaneous injection at P5 and P6, with 100 mg kg^−1^ DAPT (Calbiochem, #565770) and retinas being harvested at P7.

*PdgfbiCreER*^*T2*^; Fzr1^flox/flox^ were interbred with Fzr1^flox/flox^ (ref. 51) to generate *PdgfbiCreER*^*T2*^; Fzr1^flox/flox^ and Fzr1^flox/flox^ littermates. Pups were injected with 25 μg 4-OHT (10 mg ml^−1^) at P5 and P6 and dissected at P7.

For combined endothelial-cell-specific loss-of-function of PTEN and Jag1, we crossed PTEN^flox/flox^ (ref. 50) with Jagged1^flox/flox^ (ref. 52) and *PdgfbiCreER*^*T2*^ (ref. 26). To generate *PdgfbiCreER*^*T2*^; PTEN^flox/flox^; Jag1^flox/flox^ (PTEN^iΔEC/iΔEC^; Jag1^iΔEC/iΔEC^), *PdgfbiCreER*^*T2*^; PTEN^flox/flox^ (PTEN^iΔEC/iΔEC^) and *PdgfbiCreER*^*T2*^; Jag1^flox/flox^ (Jag1^iΔEC/iΔEC^) littermates, two different types of breeding were set up; *PdgfbiCreER*^*T2*^; PTEN^flox/flox^; Jag1^flox/flox^ were interbred with PTEN^flox/flox^; Jag1^flox/WT^ or with PTEN^flox/WT^; Jag1^flox/flox^. Cre activity and gene deletion were induced by intraperitoneal injection of 25 μg 4-OHT (10 mg ml^−1^) in all pups of the litter, at P1 and P3 and retinas were collected at P7. To assess proliferating ECs, the pups were injected intraperitoneally with 60 μl of Edu (0.5 μg^−1^ μl^−1^) 2 h before being killed. Edu was dissolved in a 1:1 ratio DMSO:PBS.

### Immunofluorescence

Eyes were fixed in 4% paraformaldehye (PFA) for 2 h at 4 °C. For PTEN staining, mice were exsanguinated by transcardiac perfusion of PBS, followed by perfusion with 4% PFA before dissecting and continuing fixing the retinas with methanol at −20 °C. Samples were rehydrated for 30 min at room temperature (RT). After washing twice in PBS, retinas were permeabilized in PBS containing 1% bovine serum albumin (BSA) and 0.3% Triton X-100 overnight (ON) at 4 °C, followed by incubation with primary antibodies (PTEN (Abcam; 1:75), pSer240/244-S6 (1:100), Erg (1:200), desmin (1:200), Hes1 (1:50) and Ki67 (1:50) in permeabilization buffer ON at 4 °C. The following day, the eyes were washed three times with PBS containing 0.1% Tween (PBT), one time in Pblec buffer (1% Triton X-100, 1 mM CaCl_2_, 1 mM MgCl_2_ and 1 mM MnCl_2_ in PBS, pH 6.8) for 30 min and then incubated for 2 h at RT or ON at 4 °C in Pblec buffer containing Alexa-conjugated secondary antibodies (1:200) and IB4 (1:300), washed three times further with PBT and flat-mounted on microscope glass slides with Mowiol. For fixed confocal laser scanning microscopy, we use a Leica SP5. Images were analysed with Image J Software and Adobe Photoshop CS5.

### Embryoid bodies

ES cells were cultured and EBs were generated, as previously described^27^. Briefly, ES cells were regularly cultured on a layer of irradiated DR4 mouse embryonic fibroblast in DMEM glutamax (Life Technologies, #61965-026) in the presence of 20% fetal bovine serum, HEPES (30 mM), sodium pyruvate (1.5 mM), monothioglycerol (1.5%) and leukaemia inhibitory factor (Chemicon#ESG1107, 123 units ml^−1^). For vascular sprouting assays, cells were cultured for two passages without feeders, depleted of leukaemia inhibitory factor and left in suspension as hanging drops. Four days after, the formed EBs were transferred to a polymerized collagen I gel with the addition of 60 ng ml^−1^ VEGF (Peprotech). The medium was changed on day 6 and every day thereafter. Overall, 70,000 WT ES cells and 10,000 PTEN^−/−^ ES cells were plated to generate EB. PTEN^−/−^ ES cells were provided in ref. 53.

### Isolation and stimulation of mECs

Mouse lungs were digested with Dispase (Life Technologies, #17105-041; 4 units ml^−1^) for 1 h at 37 °C, followed by positive selection with antimouse vascular endothelial-cadherin (Pharmingen, #555289) antibody coated with magnetic beads (Dynal Biotech, #110-35). Cells were seeded on a 12-well plate, and were coated with gelatin (0.5%) in DMEM/F12 supplemented with 20% fetal calf serum and EC growth factor (PromoCell, #C30140). After the first passage, the cells were re-purified with vascular endothelial-cadherin antibody-coated magnetic beads. Cells were cultured until passage 6. To induce gene deletion, 4-OHT (5 μM) or vehicle (ethanol) was added to the cultured medium at P4 for 96 h and the medium was replaced every other day. For Dll4 stimulation, mouse Dll4 (500 μg ml^−1^) was immobilized by coating culture dishes for 1 h at RT, followed by seeding mECs for 6, 8 or 24 h. Mouse and human Dll4 were used accordingly.

### *In vitro* measurement of mEC cell proliferation

Overall, 10^4^ mECs were plated in a 24-well plate for 48 h; 2 h before the termination of the experiment, BrdU (10 μM) was added to the medium. For Ki67 staining, cells were plated for 24 h in Dll4-coated dishes. Cells were fixed in 4% PFA for 10 min at RT, permeabilized for 10 min with TBS-T (25 mM Tris HCl pH 7.4, 150 mM NaCl, 0.5% Triton X-100), blocked with TBS-T containing 2% BSA and incubated with primary antibodies BrdU (1:100) or Ki67 (1:50) at 4 °C ON. The following day, cells were washed three times with TBS-T and incubated with Alexa-conjugated secondary antibodies for 2 h at RT. DAPI was added in the final wash. Specimens were mounted in Mowiol. Cells were visualized in a Nikon-80I microscope. For pharmacological inhibition of class I PI3K and Aurora kinase, GDC-0941 (1 μM) and VX680 (0.5 μM) were added, respectively, on plating.

### Plasmids and transfections

pRK5-Myc-PTEN, C124S pEGFP-PTEN-wt and pEGFP-PTEN-K13,289E expressing human WT, lipid phosphatase-inactive and nuclear-excluded PTEN mutants, respectively, were provided in ref. 22. All three PTEN mutants were subcloned with an N-terminal yellow fluorescent protein into a modified lentiviral vector TRIPZ. Lentiviral particles were prepared by transfecting HEK293FT cells with the TRIPZ vector of interest and the packaging vectors psPAX, VSV-G and pTAT. Viral particles in the supernatant were concentrated with Lenti-X-concentrator (Clontech). mECs of P2 or P3 from PdgfbiCreER^T2^; PTEN^flox/flox^ were infected with lentivirus expressing WT PTEN, PTEN (C124S) or PTEN (K13,289E) in the presence of viralplus transduction enhancer (Applied Biological Material #G698). For infection, mECs were plated at a density of 4 × 10^4^ per well of 12-well plate and infected with virus from 293FT cells 48 h after transfection. After 48 h post infection, mECs were re-plated and treated with 4-OHT (5 μM) to induce gene deletion for 72 h. Next, 10^4^ mECs were plated in 24-well plate for 48 h in the presence of doxycycline (4 μM); 2 h before the termination of the experiment, BrdU (10 μM) was added to the medium. Cells were then fixed in 4% PFA for 10 min at RT, permeabilized for 10 min with TBS-T (25 mM Tris HCl pH 7.4, 150 mM NaCl, 0.5% Triton X-100), blocked with TBS-T containing 2% BSA and incubated with primary antibodies BrdU (1:100) at 4 °C ON. The following day, cells were washed three times with TBS-T and incubated with Alexa-conjugated secondary antibodies for 2 h at RT. DAPI was added in the final wash. Specimens were mounted in Mowiol. Cells were visualized in a Nikon-80I microscope.

### MTS viability assay

mECs were cultured in 96-well plate (2,000 cells per 100 μl culture medium per well) in the presence of the test compounds (GDC-0941 (1 μM) and VX680 (0.5 μM)) or the respective controls for 48 h, followed by MTS assay (Promega, #G5421).

### Protein extraction and immunoblotting

mECs, human umbilical vascular ECs (HUVECs (Lonza #CC-2519)) and lungs were lysed in 50 mM Tris HCl pH 7.4, 5 mM EDTA, 150 mM NaCl, 50 mM NaF and 1% Triton X-100 supplemented with 2 mg ml^−1^ aprotinin, 1 mM pepstatin, 1 ng ml^−1^ leupeptin, 1 mM phenylmethysulfonylfluoride and 1 mM sodium orthovanadate, followed by clearance of lysates using microcentrifugation. Supernatants were resolved on a 10% SDS–PAGE gel, transferred on nitrocellulose membranes and probed with the indicated antibodies. Detection was performed by enhanced chemiluminescence. Uncropped immunoblots and larger blot areas are presented in Supplementary Fig. 8.

### qPCR analysis

qPCR was performed using the following proprietary TaqMan Gene Expression assay FAM/TAMRA primers (Applied Biosystems): Dll4 (Mm00446968_m1), Hes1 (Mm01342805_m1), Hey1 (Mm00468865_m), Nrarp (Mm00482529_m1), Ephb4 (Mm00438750_m1), Efnb2 (Mm01215897_m1), Nr2f2 (Mm00772789_m1) and Hprt (Mm00446968_m1). The levels of PTEN mRNA were measured using SYBR Green I Master (Roche, #04.887.352.001) in the LightCycler480 system. Primers used are as follow: forward (5′-GTTTACCGGCAGCATCAAAT-3′) and reverse (5′-CCCCCACTTTAGTGCAC-3′).

### Luciferase assays

Reporter assays in HUVECs were performed with the Dual Luciferase Assay System (Promega, #E1910) and a LUMAT LB 9507 luminometer (BERTHOLD Technologies). HUVECs were grown to 60–70% confluence in endothelial basal medium (EGM; Lonza #CC-3124) and co-transfected (Trans Pass V Reagents (New England Biolabs, # M2558S)) with V5-NICD (ref. 54), PTEN-luciferase reporter (pGL3 PTEN HindIII-NotI) construct^37^ and the constitutive Renilla luciferase reporter pGL4.74hRluc/TK (Promega). Twenty-four hours after, HUVECs were lysed and reporter assays performed according to the manufacturers' protocol. To induce Notch activity with Dll4, transfected HUVECs were re-plated on Dll4-coated dishes 6 h after plasmid infection. Luciferase activity was measured after an additional 24 h. To inhibit Notch signalling, cells were pretreated for at least 1 h before stimulating with Dll4 with 0.08 μM DBZ ((S,S)-2-[2-(3,5-Difluorophenly)acetylamino]-N-(5-methyl-6-oxo-6,7-dihydro-5H-dibenzo[b,d]azepin-7-yl)propionamide).

### ChiP assay

To analyse the binding sites for RBPJ located in the PTEN proximal promoter, we used the Genomatix software. For analysis, the gene bank sequence used was NG_007466.2, which contains the promoter sequence AF406618.1. Three putative RBPJ-binding sites located at −1,914, −1,492 and −1,132 positions relative to transcription initiation site^37^ were identified. ChiP assay was performed as previously described^55^. Briefly, chromatin was isolated from HUVECs stimulated for 2 h with vehicle or Dll4 (500 ng ml^−1^). Crosslinked chromatin was sonicated for 10 min, to medium-sized powder particles, at 0.5-min intervals, with a Bioruptor (Diagenode) and precipitated with anti-NCID or control IgG. After crosslinkage reversal, DNA was used as a template for PCR. qPCR was performed with SYBR Green I Master (Roche, #04.887.352.001) in the LIghtCycler480 system. Primers used are described in Supplementary Table 1.

## Additional information

**How to cite this article**: Serra, H. *et al*. PTEN mediates Notch-dependent stalk cell arrest in angiogenesis. *Nat. Commun.* 6:7935 doi: 10.1038/ncomms8935 (2015).

## Supplementary Material

Supplementary Information Supplementary Figures 1-8 and Supplementary Table 1
