# Supplementary material for: PTEN mediates Notch-dependent stalk cell arrest in angiogenesis
Source: Nat Commun. 2015 Jul 31;6:7935. doi: 10.1038/ncomms8935 (PMC5426521; doi:10.1038/ncomms8935)
Supplement: Supplementary Information — Supplementary Figures 1-8 and Supplementary Table 1 [file ncomms8935-s1.pdf]

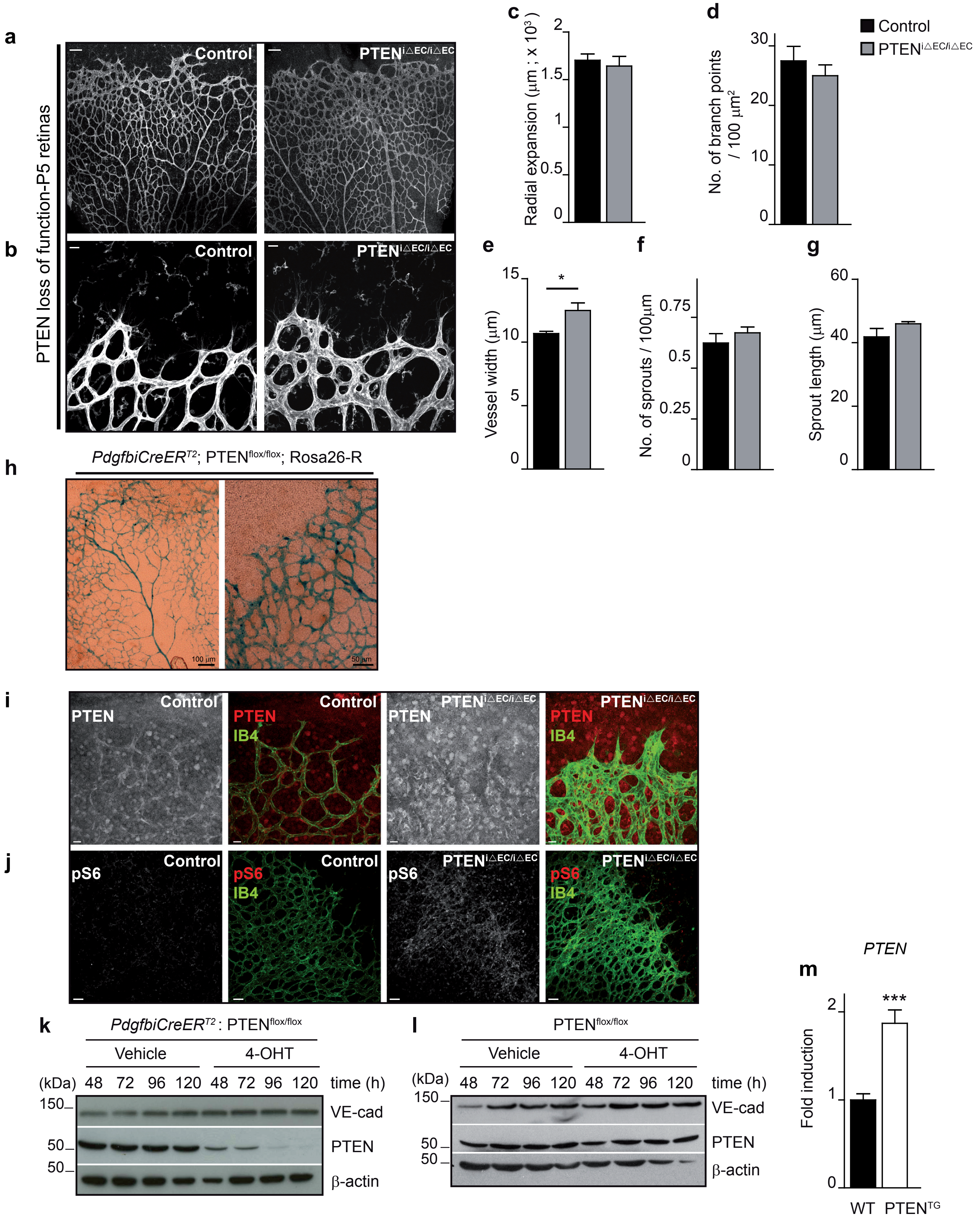

Supplementary Figure 1. Retinal angiogenesis upon endothelial specific PTEN deletion. (**a**,**b**) Whole mount visualization of blood vessels by IB4 staining of control and PTEN$^{iΔEC/iΔEC}$ littermates at P5. Scale bars, 100 μm (**a**) and 20 μm. (**b**) Quantitative analysis of the retinas shown in **a** and **b**. (**c**) Radial expansion of blood vessels (n≥7). (**d**) Vascular branch points per unit area (n=4). (**e**) Vessel width per unit area (n≥4). (**f**) Number of sprouts per vascular front length (n≥3). (**g**) Sprout length from the tip to the base of the sprout (n≥3). (**h**) Cre expression detected by X-gal staining (blue) in P7 retinas of *PdgfbiCreER^{T2}*; PTEN$^{flox/flox}$; Rosa26-R pups (n=3). (**i**,**j**) Cre activity results in deletion of the floxed PTEN allele shown by lack of PTEN (**i**) and increased pS6 (**j**) immunostaining in P7-P9 PTEN$^{iΔEC/iΔEC}$ retinas compared to control retinas (n=2). Scale bars, 20 μm (**i**) and 50 μm (**j**). 4-OHT was administered at P1 and P2 in **a**-**j**. (**k**,**l**) Exponential growing *PdgfbiCreER^{T2}*; PTEN$^{flox/flox}$ (**k**) and PTEN$^{flox/flox}$ (**l**) mECs were incubated with vehicle or 4-OHT for 48 h, 72 h, 96 h or 120 h, followed by immunoblotting using the indicated antibodies. Molecular weight marker (kDa) is indicated. (**m**) mRNA levels of PTEN in WT and PTEN^{TG} P7 retinas (n≥5). Errors bars are s.e.m. *P*-values *p<0.05, and ***p<0.001 were considered statistically significant. Statistical analysis was performed by nonparametric Mann Whitney's test.

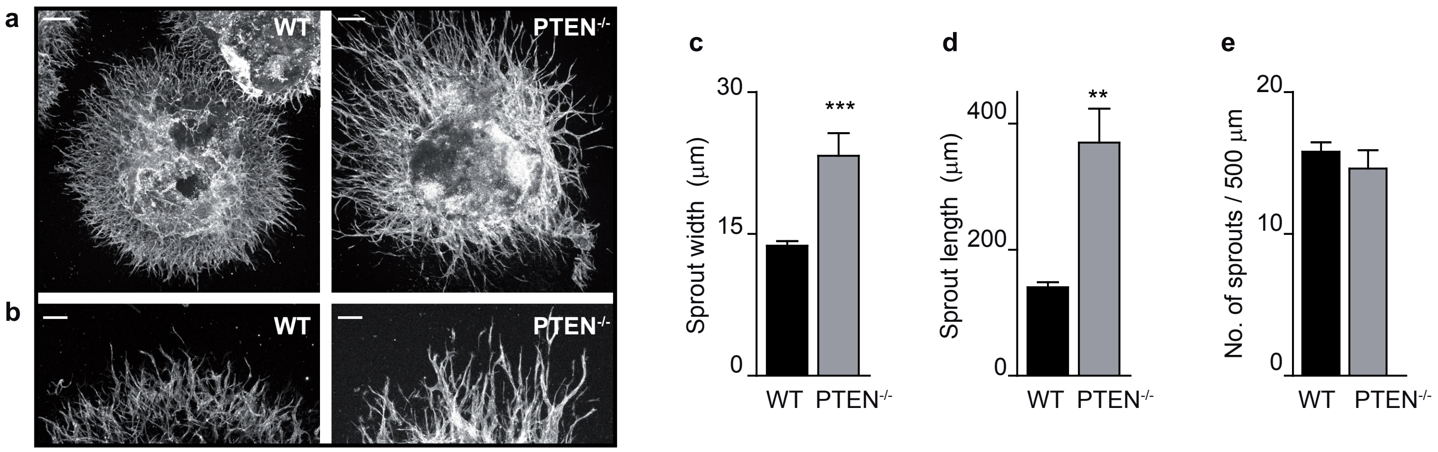

Supplementary Figure 2. Inactivation of PTEN leads to defective sprouting angiogenesis. (**a**,**b**) Representative images of WT and PTEN$^{-/-}$ embryoid bodies stained with CD31 (green). Scale bars, 200 µm (**a**) and 150 µm (**b**). (**c**-**e**) Quantitative analysis of embryoid bodies shown in **a** and **b**. (**c**) Sprouts width (n≥6). (**d**) Sprout length (n≥6). (**e**) Number of sprouts (n≥6). Errors bars are s.e.m. *P*-values **p<0.01, and ***p<0.001 were considered statistically significant. Statistical analysis was performed by nonparametric Mann Whitney's test.

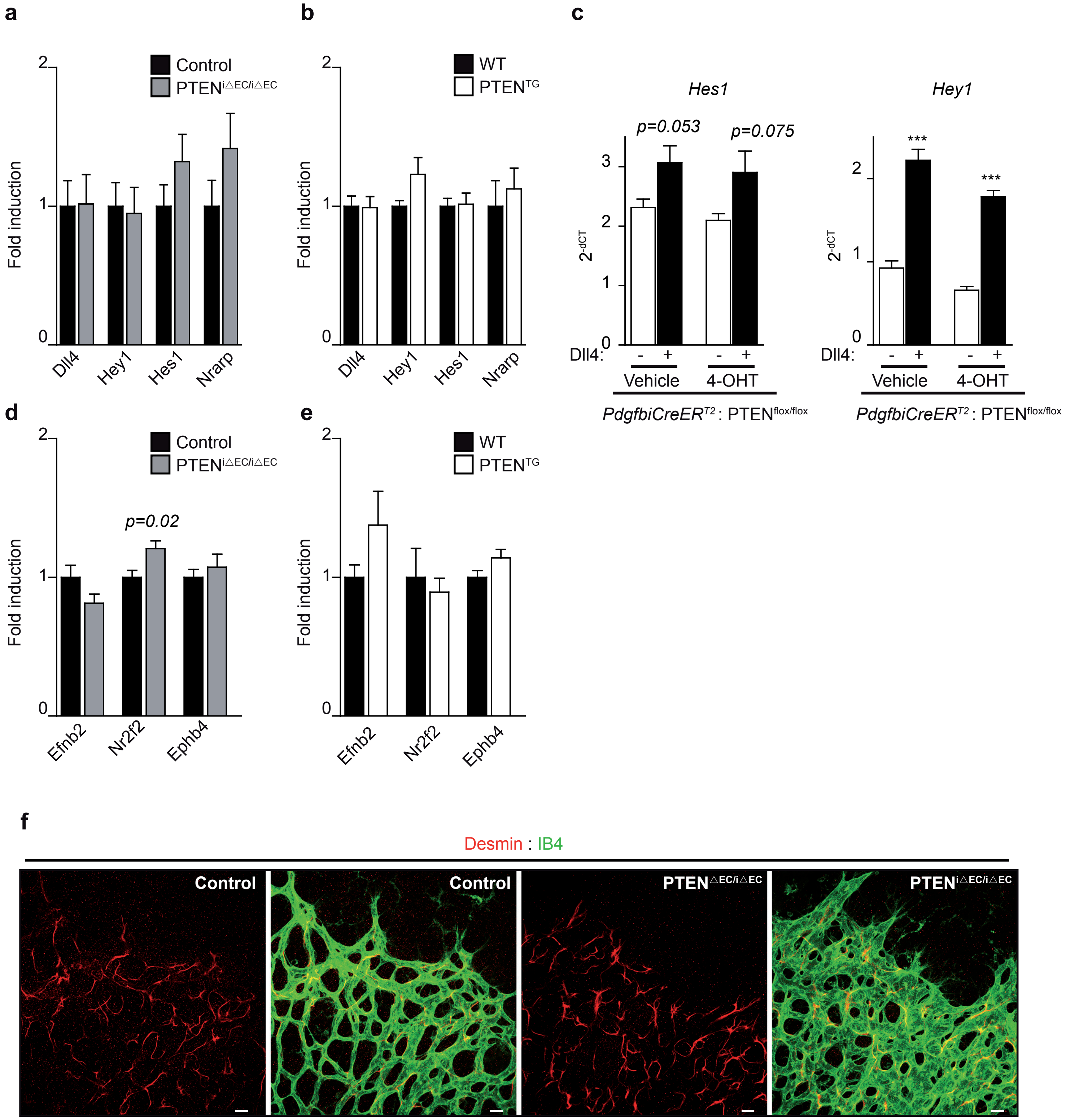

Supplementary Figure 3. PTEN does not regulate Notch target genes. (**a**,**b**) qPCR of Dll4, Hey1, Hes1 and Nrarp mRNA expression in the retinas of control and $PTEN^{i\Delta EC/i\Delta EC}$ littermates at P7 (n≥5) (**a**) and WT and $PTEN^{TG}$ (n≥5) (**b**). (**c**) Control and $PTEN^{i\Delta EC/i\Delta EC}$ mECs were stimulated with Dll4 for 6 h, followed by qPCR analysis of Hes1 and Hey1 (n=4). (**d**,**e**) qPCR of Efnb2, Nr2f2, and Ephb4 mRNA expression in the retinas of control and $PTEN^{i\Delta EC/i\Delta EC}$ littermates at P7 (n≥6) (**d**) and WT and $PTEN^{TG}$ (n≥5) (**e**). mRNA levels were normalized by Hprt in **a**, **b**, **c**, **d** and **e**. (**f**) IB4 (Green) and desmin (Red) staining of control and $PTEN^{i\Delta EC/i\Delta EC}$ littermate retinas at P7 (n=3). Scale bars, 20 μm. Errors bars are s.e.m. *P*-value ***p<0.001 were considered statistically significant. Statistical analysis was performed by nonparametric Mann Whitney's test.

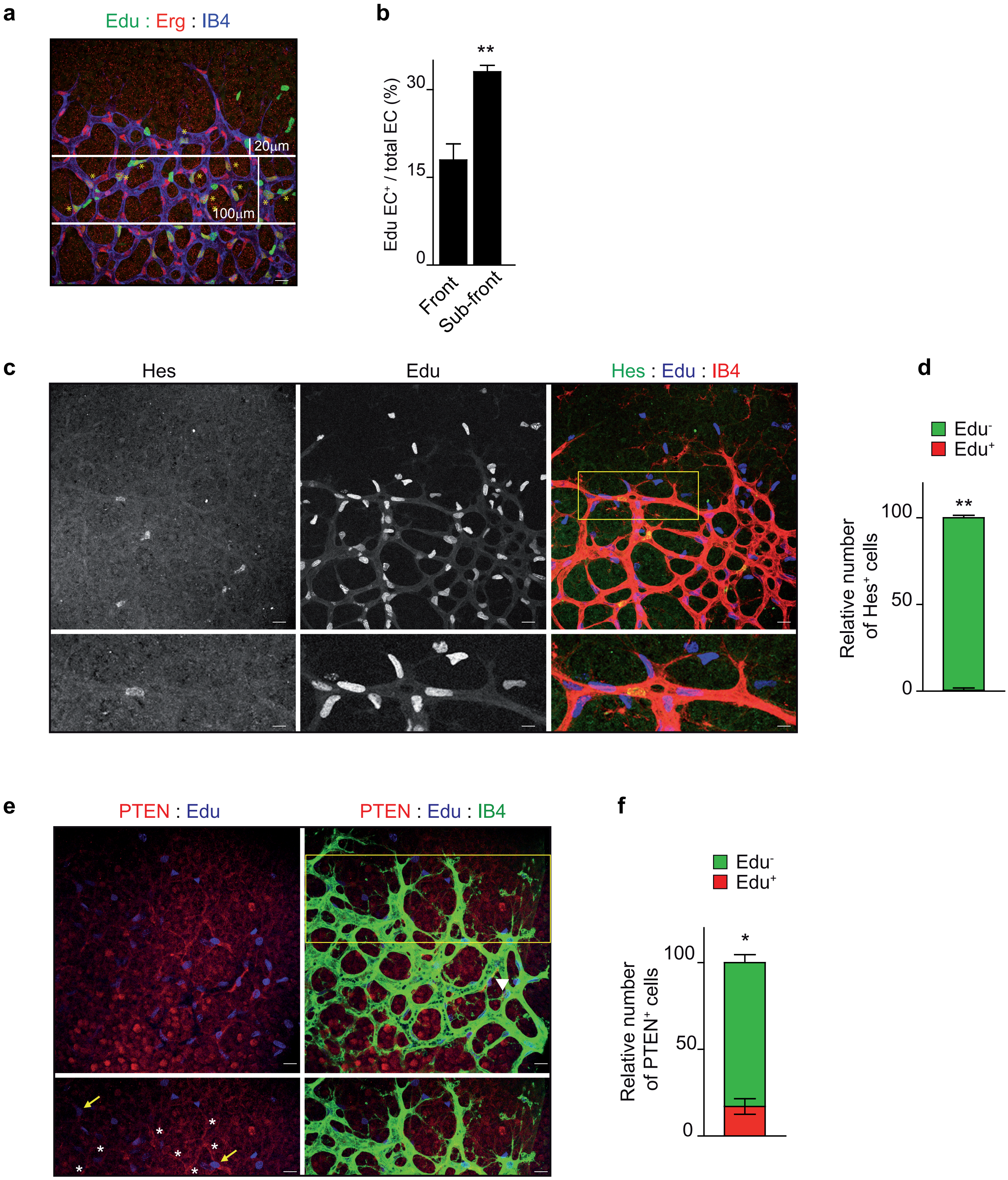

Supplementary Figure 4. Notch negatively regulates EC proliferation. (**a**) WT mice were injected with Edu 2 h before isolating the retinas, followed by triple staining with IB4 (Blue), Erg (Red) and Edu (Green). Number of proliferative ECs was quantified at the sprouting front (20 µm from the sprouting front line) and at the sub-front area (comprise the area enclosed 100 µm below the sprouting front). Scale bars, 20 µm. (**b**) Quantification of Edu positive cells at the sprouting front and at the sub-front area (n=8). (**c**) P6 WT mice were injected with Edu 2 h before isolating the retinas, followed by triple staining with IB4 (Red), Hes1 (Green) and Edu (Blue). Scale bars, 20 µm. Islets show higher magnification of selected regions shown below. Scale bars, 10 µm. (**d**) Quantification of Hes1/ Edu positive cells relative to Hes1 positive/ Edu negative cells in the vascular plexus (n=6). (**e**) P6 WT mice were injected with Edu two hours before isolating the retinas, followed by triple staining with IB4 (Green), PTEN (Red) and Edu (Blue). Islets show higher magnification of selected regions shown below. Yellow arrows and white asterisk indicate high PTEN expression/ Edu positive ECs and high PTEN expression/ Edu negative ECs respectively. White arrowhead indicates a pericyte. Scale bars, 20 µm. (**f**) Quantification of PTEN expression levels vs. Edu positivity at the vascular front (n=4). Errors bars are s.e.m. *P*-values *p<0.05, and **p<0.01 were considered statistically significant. Statistical analysis was performed by nonparametric Mann Whitney's test.

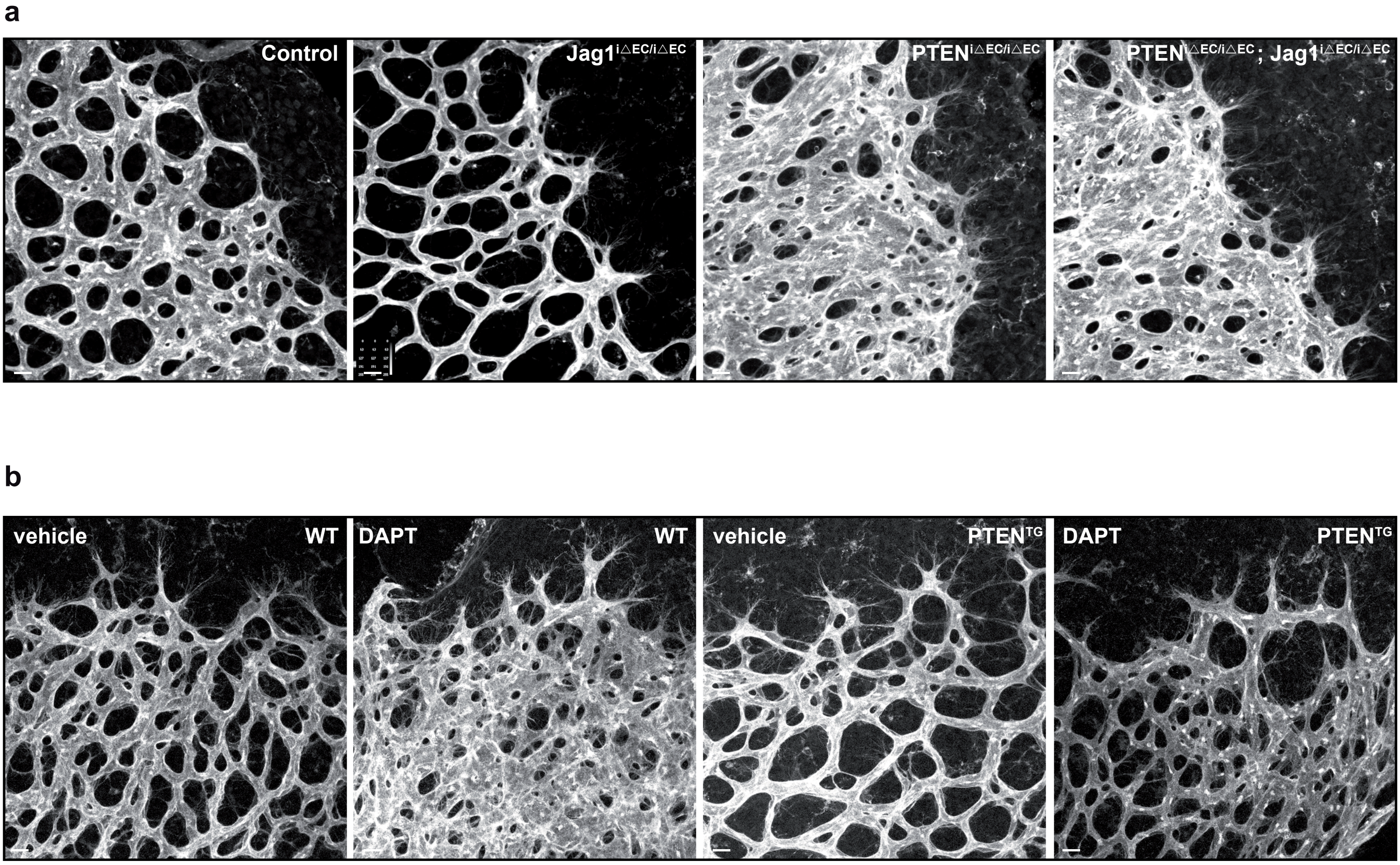

Supplementary Figure 5. PTEN operates downstream of Notch *in vivo.* (**a**) Whole mount visualization of blood vessels by IB4 staining of control, Jag1$^{i\Delta EC/i\Delta EC}$, PTEN$^{i\Delta EC/i\Delta EC}$ and PTEN$^{i\Delta EC/i\Delta EC}$; Jag1$^{i\Delta EC/i\Delta EC}$ littermates at P7. (**b**) Whole mount visualization of blood vessels by IB4 staining of WT and PTEN^{TG} P7 retinas treated with vehicle or DAPT (100 mg kg^{-1}). Scale bars, 20 μm.

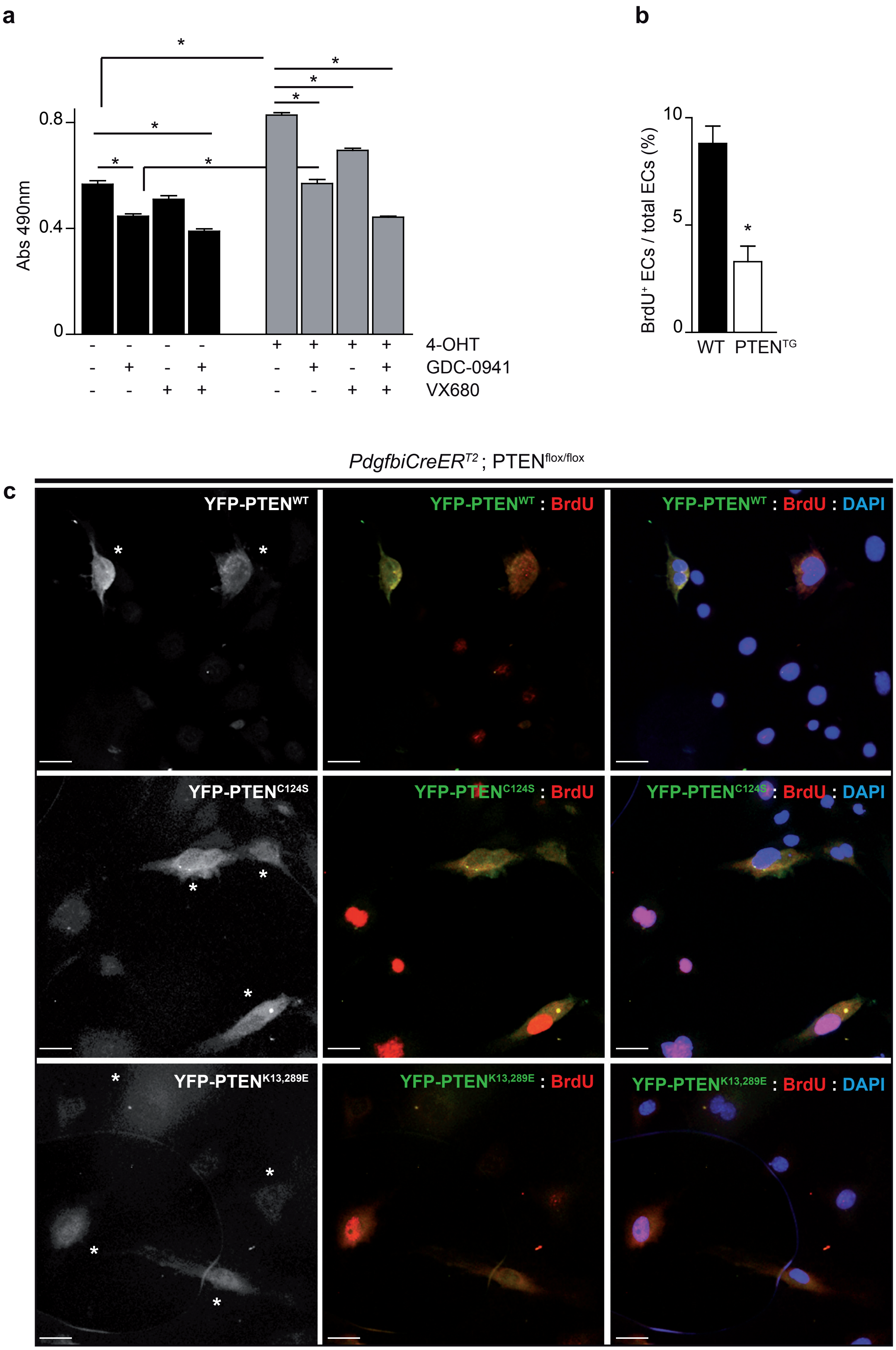

Supplementary Figure 6. PTEN regulates endothelial cell proliferation *in vitro.* (**a**) Effect of pan PI3K inhibitor GDC-0941 (1 μM) and VX680 (0.5 μM) on control and $PTEN^{i\Delta EC/i\Delta EC}$ mEC proliferation was assessed by MTS assay after treatment for 48 h. One representative of three experiments is shown. (**b**) Quantification of proliferation of WT and $PTEN^{TG}$ mECs plated for 48 h, pulsed with BrdU for 2 h, and subjected to immunostaining analysis. Data shown are means of four independent experiments. (**c**) Immunoflorescence analysis of YFP tagged wild-type PTEN, $PTEN^{C124S}$, and $PTEN^{K13,289K}$, stained with BrdU (red) and DAPI. Images are representative staining of quantification shown in Fig. 5**g**. Scale bars, 25 μm. White asterisk indicate strong YFP positive cells. Errors bars are s.e.m. *P*-values *$p<0.05$, were considered statistically significant. Statistical analysis was performed by nonparametric Mann Whitney's test.

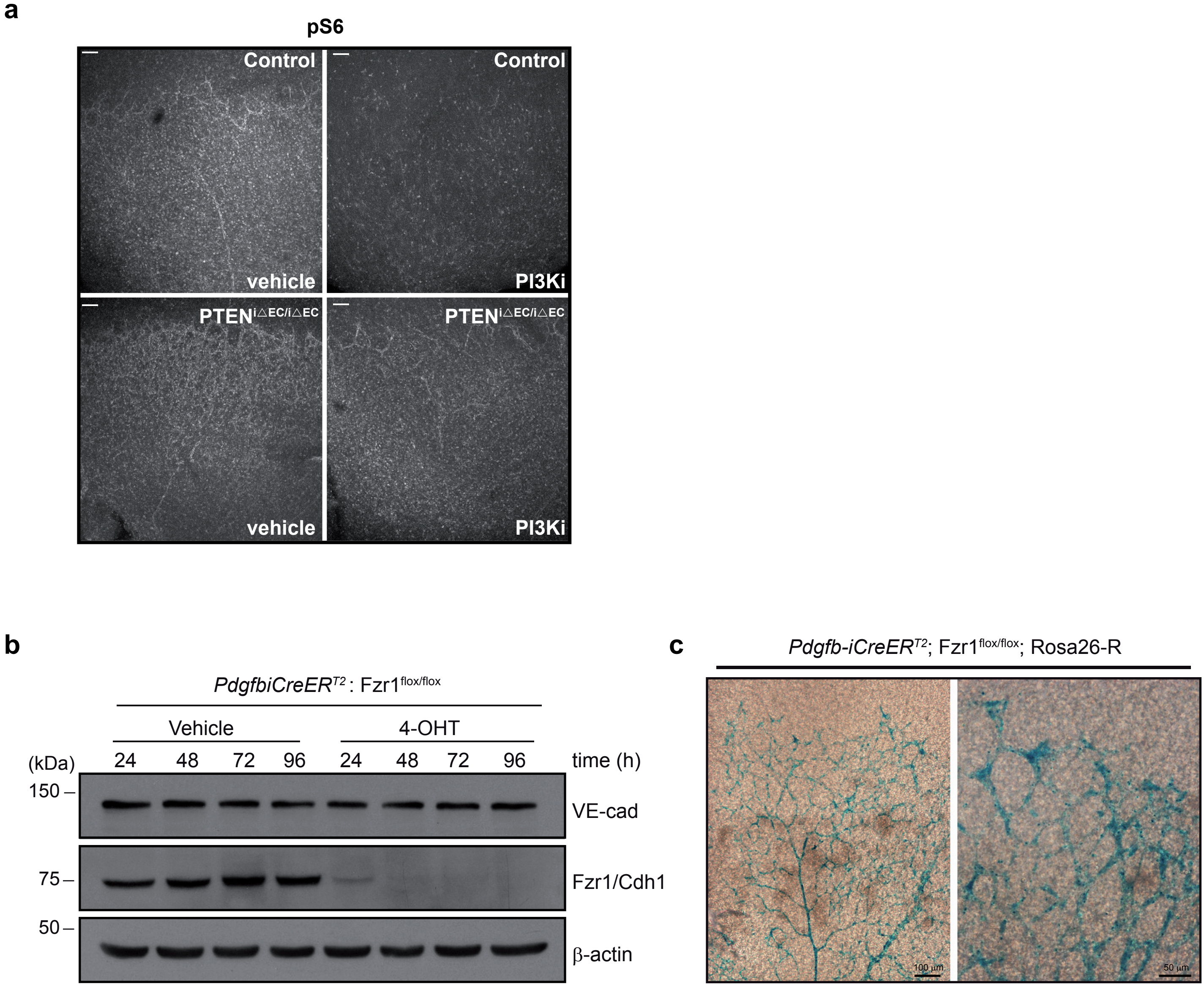

Supplementary Figure 7. Lipid phosphatase dependent and independent roles of PTEN regulate angiogenesis. (**a**) pS6 staining of images shown in Fig. 6 **a**. Scale bars, 100 µm. (**b**) Exponential growing *PdgfbiCreER*^{T2}; Fzr1$^{flox/flox}$ and Fzr1$^{flox/flox}$ mECs were incubated with vehicle or 4-OHT for 24 h, 48 h, 72 h, and 96h followed by immunoblotting using the indicated antibodies (n=2). Molecular weight marker (kDa) is indicated. (**c**) Cre expression detected by X-gal staining (blue) in P7 retinas of *PdgfbiCreER*^{T2}; Fzr1$^{flox/flox}$; Rosa26-R pups. 4-OHT was administered at P5 and P6 (n=3).

SFig. 1k

VE-cad

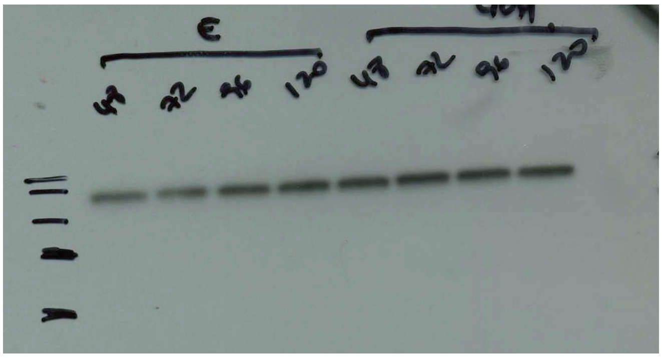

PTEN

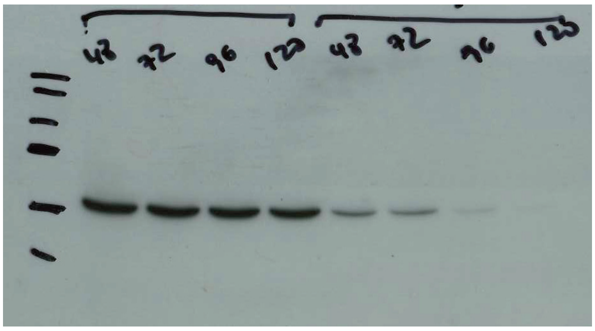

β-actin

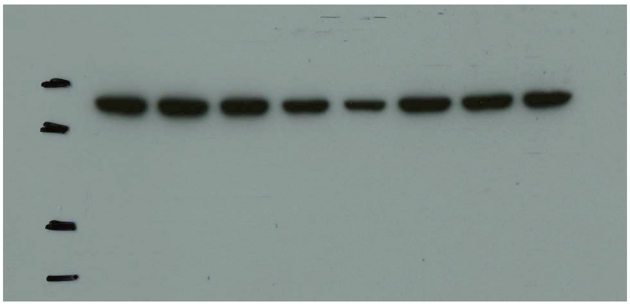

SFig. 1l

VE-cad

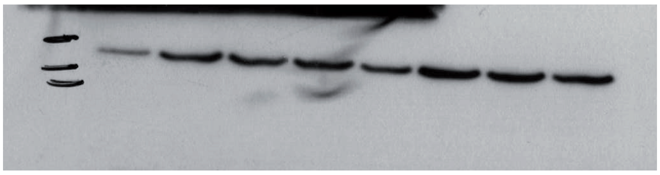

PTEN

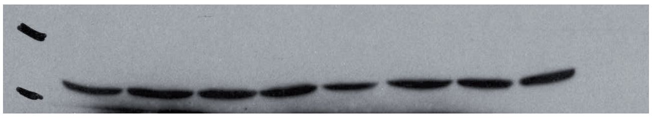

β-actin

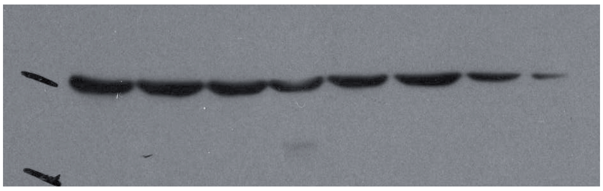

Fig. 3b-mECs

Plk1

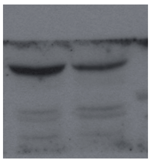

Cyclin A

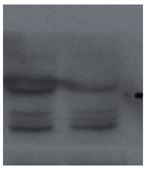

Aurora

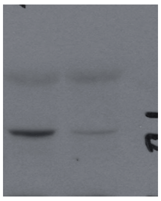

Geminin

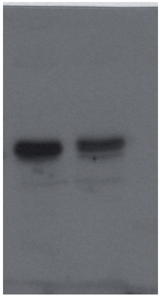

Cyclin D

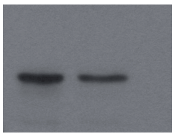

Tubulin

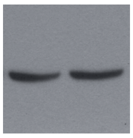

Supplementary Figure 8. Uncropped western blots related to Fig. 3, 5 and Supplementary 1, 7

Fig. 3b-HUVECs

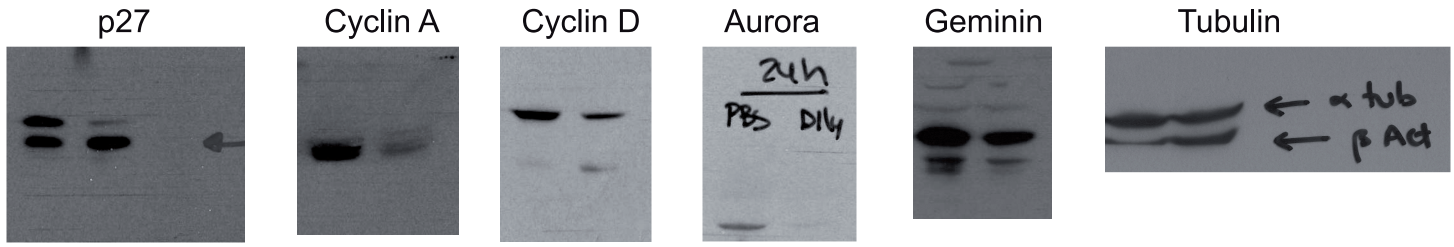

Fig. 3d

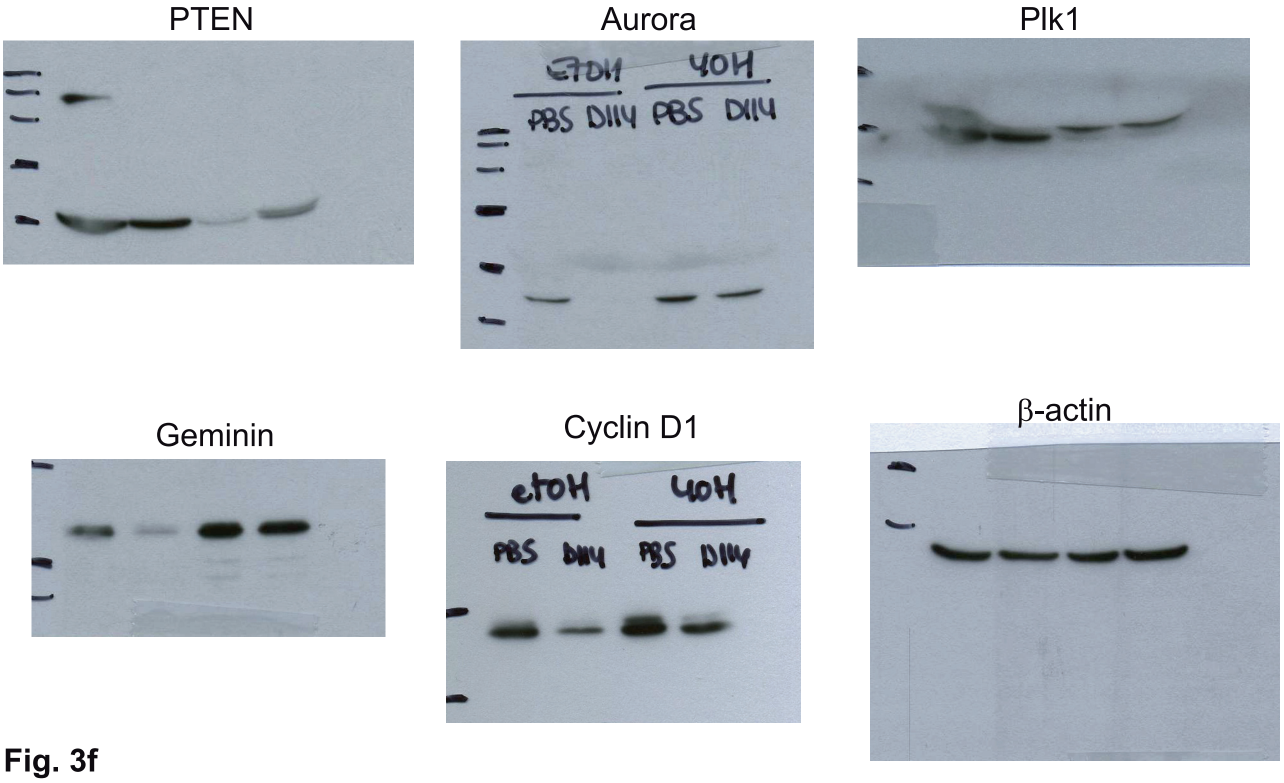

Fig. 3f

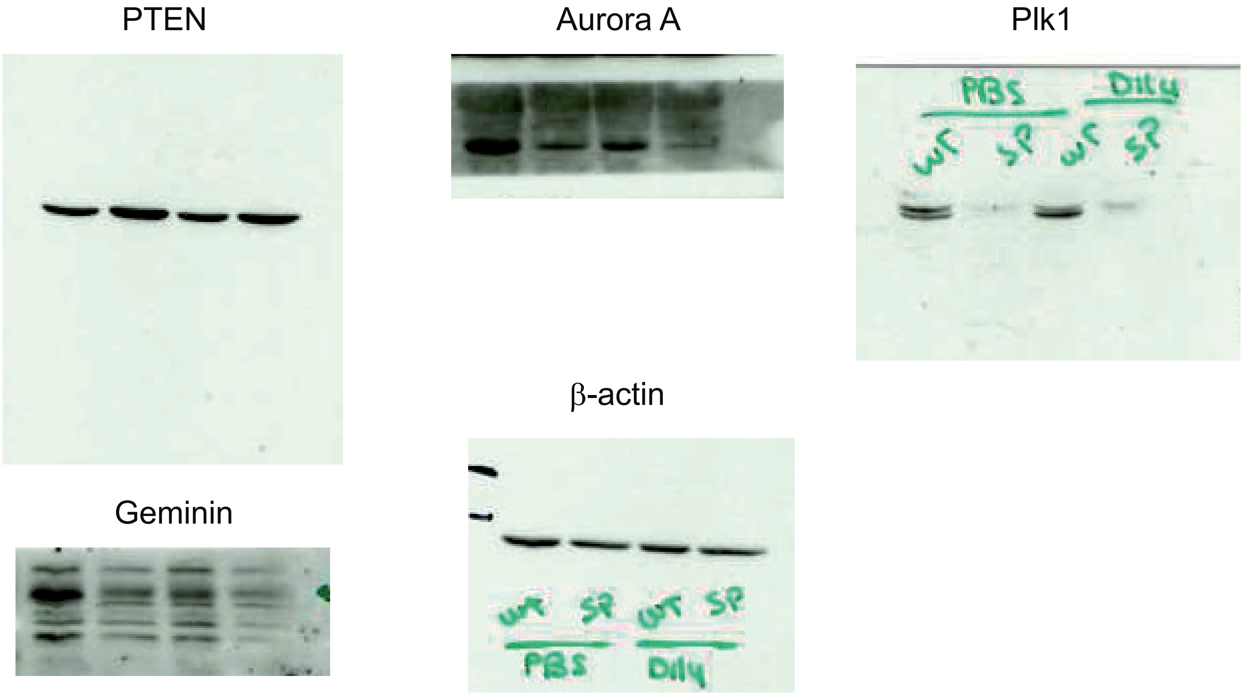

Supplementary Figure 8. Continued

Fig. 3j

PTEN

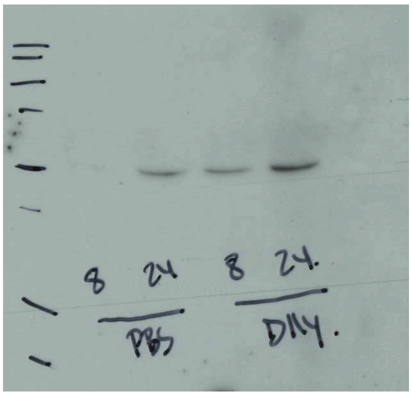

β-actin

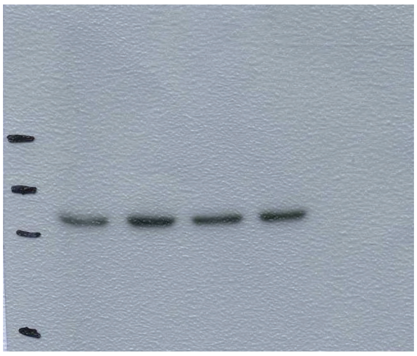

Fig. 3k

PTEN

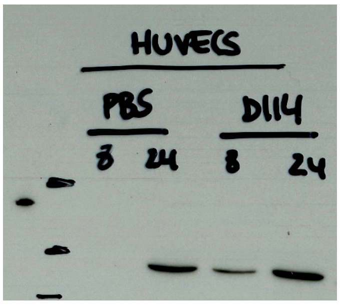

β-actin

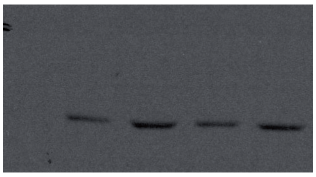

Fig. 3l

PTEN

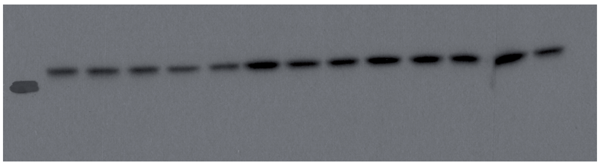

Hes1

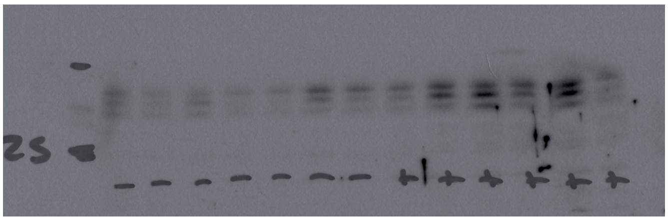

β-actin

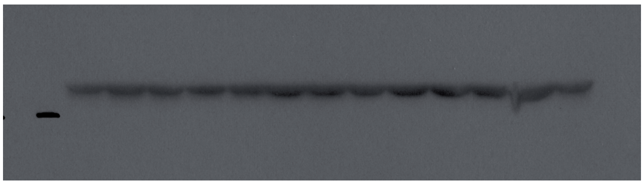

Supplementary Figure 8. Continued

Fig. 5b

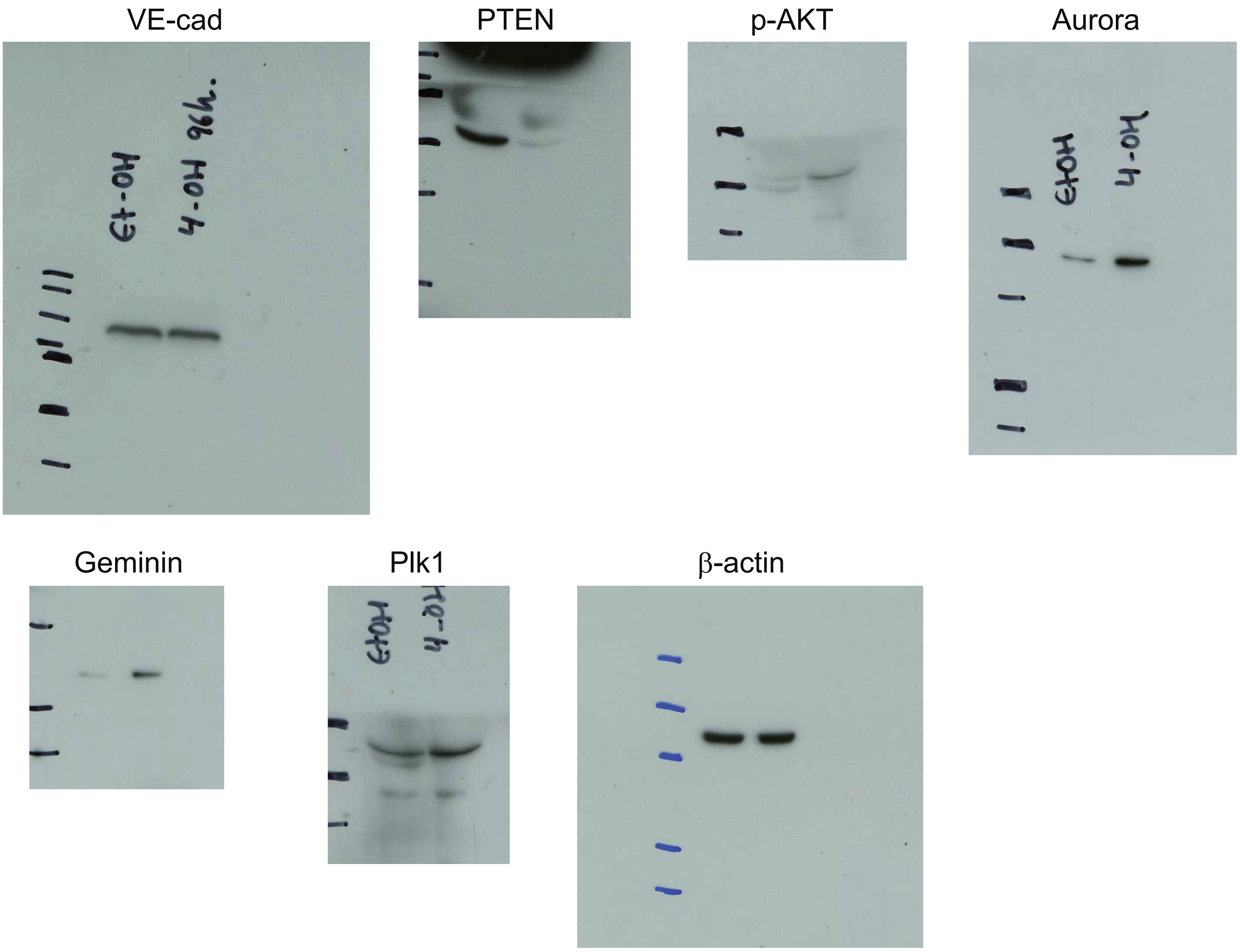

Fig. 5c

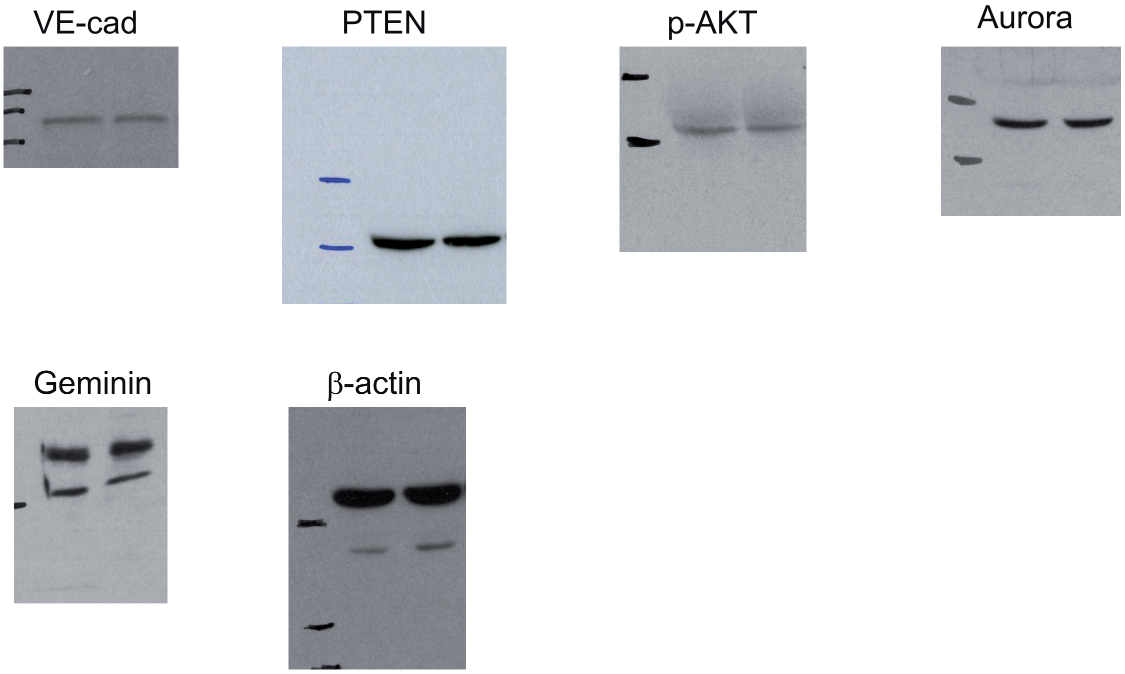

Supplementary Figure 8. Continued

Fig. 5d

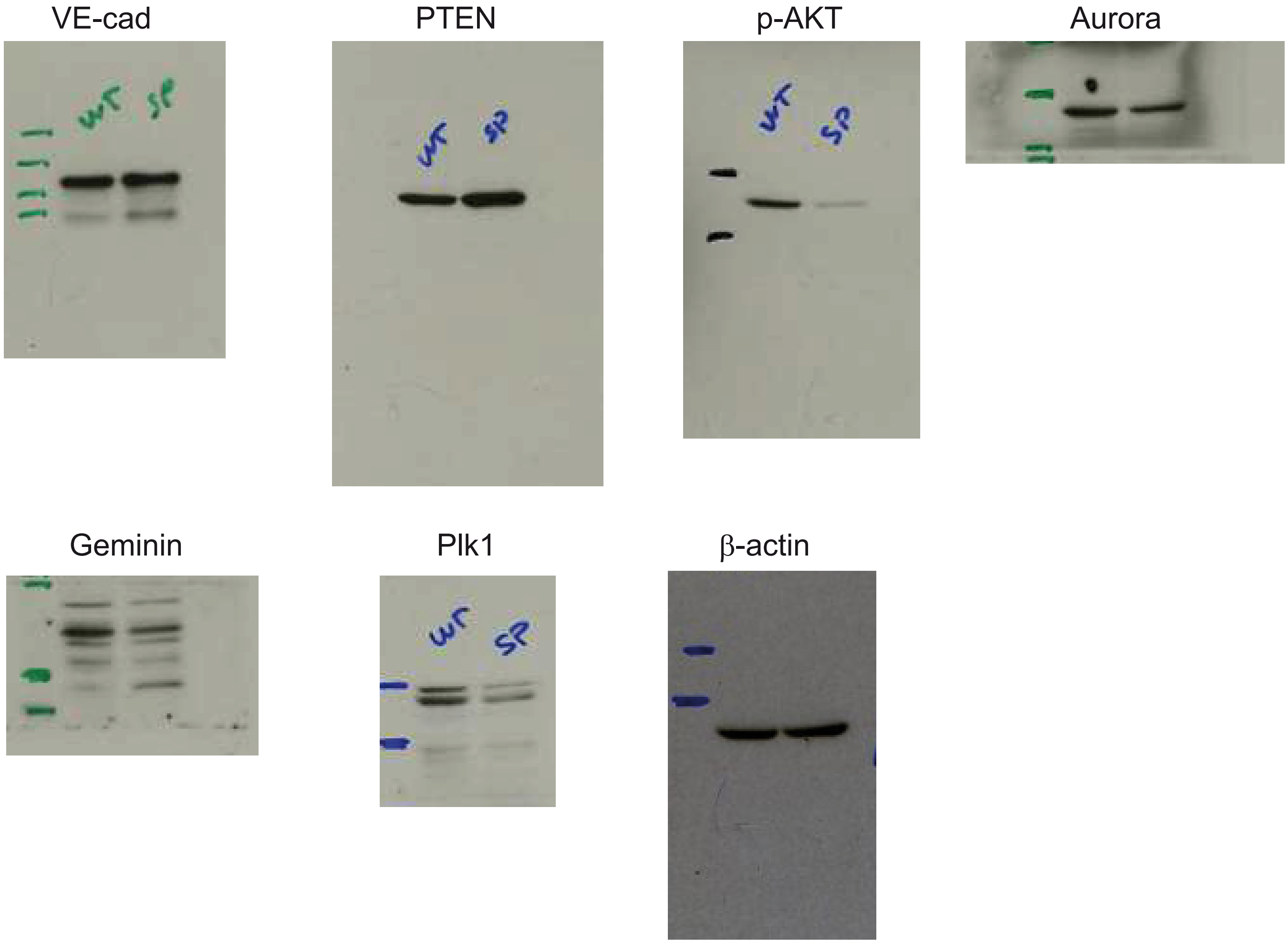

Fig. 5e

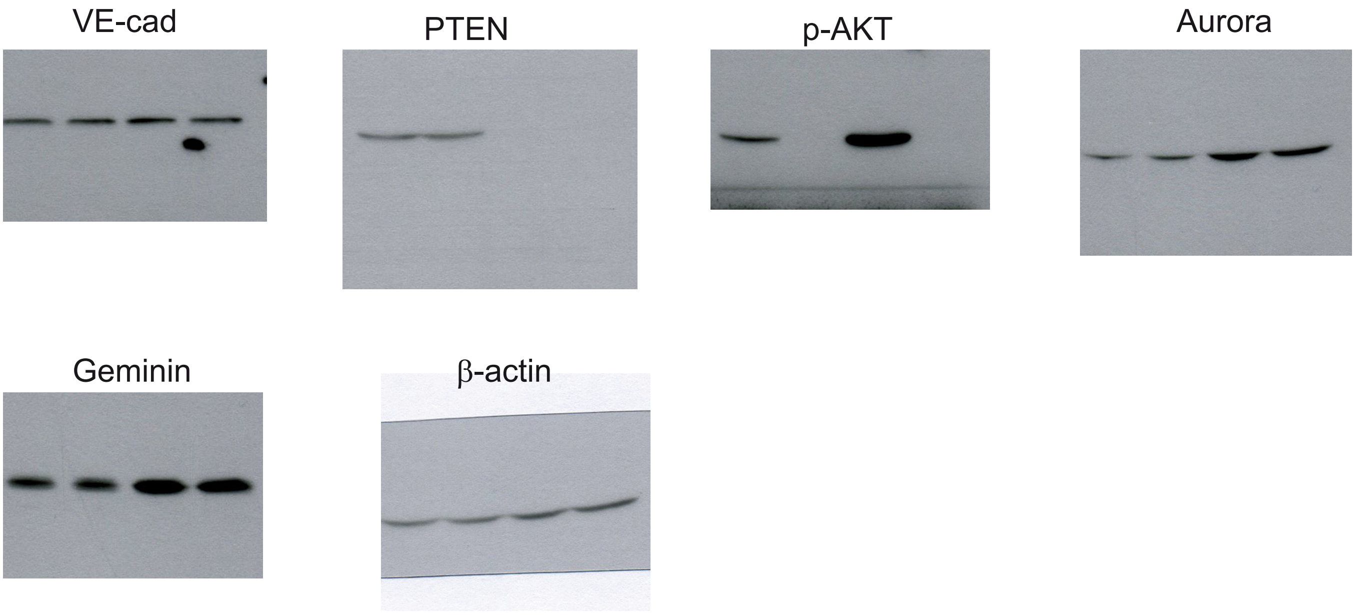

Supplementary Figure 8. Continued

SFig. 7b

VE-Cad

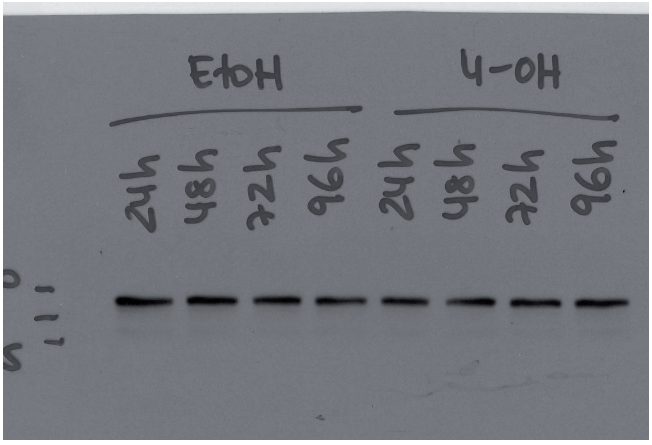

Fzr1/Cdh1

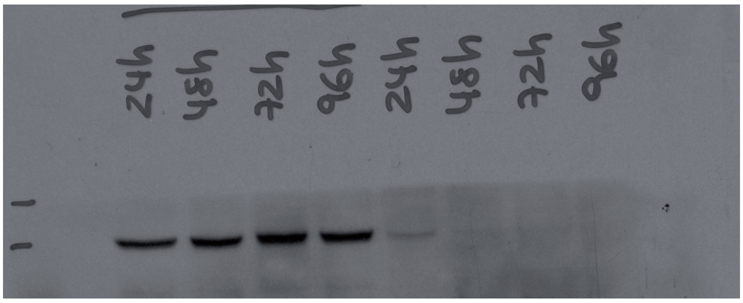

β-actin

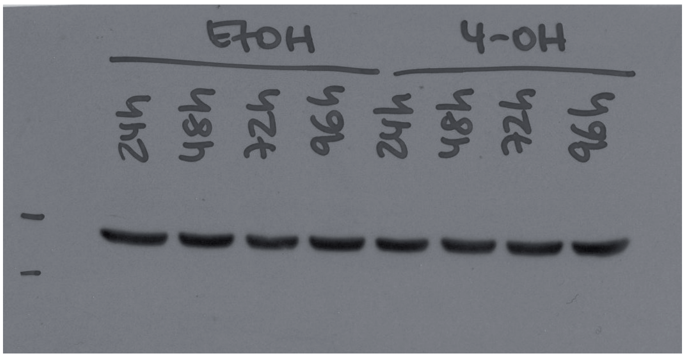

Supplementary Figure 8. Continued

Supplementary Table 1. Human ChiP primers

Name	Forward Primer	Reverse Primer
1 **(-2320 / -2181)**	5'-GGTTGTTAACTCCTGCACAGC-3'	5'-AAAGCCACAGGCCATTACC-3'
2 **(-2276 / -2136)**	5'-GCAAAGTTTTATGGAGCCTCA-3'	5'-GCAGGCAACCTCTGAAGACT-3'
3 **(-2000 / -1871)**	5'-TGCTTCAACCTAGGTCTCGTT-3'	5'-CGACCCGCTTAAAGAGAATG-3'
4 **(-1595 / -1418)**	5'-GCAGGAGATACCCTCAAGCA-3'	5'-GCAAGCCAAAGGACTGAGAC-3'
5 **(-1395 / -1286)**	5'-CACCAGTTTGGGGACTCTCT-3'	5'-GGGGAACTGGTTACACAAGC-3'
6 **(-1223 / -1092)**	5'-CAGGAAGGGTTGGGGTTC-3'	5'-TCACGTGTGTCCCTAGTTGG-3'
7 **(-803 / -658)**	5'-GGGAAAGATGCTCGACTCTC-3'	5'-ACGTGAACACATAGCCGTTG-3'
